# Evaluating the discoverability of supporting research materials in ClinicalTrials.gov for US federally funded COVID-19 clinical studies

**DOI:** 10.5195/jmla.2024.1799

**Published:** 2024-07-01

**Authors:** Paije Wilson, Vojtech Huser

**Affiliations:** 1 paije.wilson@wisc.edu, Health Sciences Librarian, University of Wisconsin-Madison School of Medicine and Public Health, Madison, WI; 2 v.huser@gwu.edu, Adjunct Professor of Clinical Research and Leadership, The George Washington University School of Medicine & Health Sciences, Washington, D.C.

**Keywords:** Clinical studies, COVID-19, Data sharing, clinicaltrials.gov, research transparency, discoverability

## Abstract

**Objective:**

The objective of this study was to evaluate the discoverability of supporting research materials, including supporting documents, individual participant data (IPD), and associated publications, in US federally funded COVID-19 clinical study records in ClinicalTrials.gov (CTG).

**Methods:**

Study registration records were evaluated for (1) links to supporting documents, including protocols, informed consent forms, and statistical analysis plans; (2) information on how unaffiliated researchers may access IPD and, when applicable, the linking of the IPD record back to the CTG record; and (3) links to associated publications and, when applicable, the linking of the publication record back to the CTG record.

**Results:**

206 CTG study records were included in the analysis. Few records shared supporting documents, with only 4% of records sharing all 3 document types. 27% of records indicated they intended to share IPD, with 45% of these providing sufficient information to request access to the IPD. Only 1 dataset record was located, which linked back to its corresponding CTG record. The majority of CTG records did not have links to publications (61%), and only 21% linked out to at least 1 results publication. All publication records linked back to their corresponding CTG records.

**Conclusion:**

With only 4% of records sharing all supporting document types, 12% sufficient information to access IPD, and 21% results publications, improvements can be made to the discoverability of research materials in federally funded, COVID-19 CTG records. Sharing these materials on CTG can increase their discoverability, therefore increasing the validity, transparency, and reusability of clinical research.

## INTRODUCTION

The COVID-19 pandemic has transformed the clinical research landscape, with one such effect being the rapid generation of COVID-19 clinical studies [1–3]. As of May 19, 2023, the classic version of ClinicalTrials.gov (CTG), a clinical study registry maintained by the US National Library of Medicine (NLM), retrieved well over 9,000 results with its COVID-19 filter [4]. While this rapid influx in clinical studies has generated groundbreaking discoveries relating to the prevention and treatment of COVID-19, it has also raised concerns relating to the quality of these studies and, consequently, the reliability of their findings [2, 5–7].

Sharing the research materials associated with a study, including full datasets, publications, and supporting documents (i.e., protocols, informed consent forms, and statistical analysis plans), increases the validity, transparency, reproducibility, and overall utility of study results, and can help to foster public trust in clinical research findings [2, 8–15]. Many organizations, such as the National Institutes of Health (NIH), the Food and Drug Administration (FDA), and the International Committee of Medical Journal Editors (ICMJE), require and/or encourage the sharing of datasets, publications, and supporting documents deriving from clinical studies [12, 16–18], with many additional organizations calling for the release of these materials during the COVID-19 pandemic [19–21]. The release of the Office of Science and Technology Policy's 2022 memorandum, “Ensuring free, immediate, and equitable access to federally funded research” (aka the Nelson Memo), additionally calls for the public availability of research materials deriving from all federally funded research [22].

While these policies are certainly helpful in encouraging the sharing of clinical research materials, the utility of these materials is limited if they are not discoverable for unaffiliated researchers (i.e., researchers not affiliated with the clinical studies). CTG is a publicly accessible clinical study registry that facilitates the discovery of clinical studies and their associated materials [23]. CTG includes both interventional and observational studies and includes sections in each study record that allow for the sharing of (among other things) supporting documents; individual participant data (IPD) sharing plans; and publications associated with the clinical study [24]. Due to its comprehensiveness and accessibility as a clinical study information discovery tool, CTG has been utilized by multiple studies in determining the extent to which clinical studies share their research materials; however, few studies have evaluated this within the context of COVID-19, with the exceptions of Rodgers et al., which examined availability of summative results and results publications [5]; Li et al. and Larson et al., which both examined IPD sharing plans [8, 9]; and Huser & Mayer, which examined availability and cross-linking of results publications to CTG records [25]. Even fewer, if any, studies have cumulatively examined the availability of supporting documents, associated publications, and information relating to IPD access in COVID-19 CTG records, nor done a granular analysis into how this information is linked in and, in the cases of publications and IPD, cross-linked back to the CTG records. Such information can give greater insight into the current sharing practices of these materials on CTG and highlight the discoverability (or lack thereof) of these materials.

The objective of this study was to examine US federally funded COVID-19 clinical study records in CTG, specifically studies that contained at least 200 participants, to evaluate (1) links to supporting documents; (2) information on how unaffiliated researchers may access IPD and, when applicable, the linking of the IPD record back to the CTG record; and (3) links to associated publications and, when applicable, the linking of the publication record back to the CTG record. The data from this research will provide insight into the sharing practices and discoverability of supporting research materials from US federally funded COVID-19 clinical studies; contribute to discussions relating to the transparency of clinical study research; and inform librarians and the clinical investigators they serve as they prepare to meet federal sharing policies to make their research materials discoverable, accessible, and more transparent.

## METHODS

The authors decided to focus on federally funded studies due to the many policies that encourage sharing of federally funded research materials [12, 17, 18], and limited studies to those containing at least 200 participants for the sake of a convenience sample manageable for the time constraints of the project.

To facilitate understanding of commonly used terms in this paper the authors have provided a glossary in Appendix A. The authors have also provided Appendix B, which lists each of the data items collected for this study and screenshots of where the data items were collected from each record.

To isolate federally funded COVID-19 studies in ClinicalTrials.gov (CTG), a combination of the COVID-19 filter (i.e., the link to “See listed clinical studies related to coronavirus disease (COVID-19)”) and the Funder Type filters for “NIH” and “Other US Federal Agency” (on the results page of CTG) were used in the classic version of CTG, resulting in a total of 326 CTG records. Results were exported as a CSV on June 6, 2022, and saved as an Excel file.

Excel was used to filter out studies containing fewer than 200 participants, being determined by the number in the “Enrollment” column. This resulted in a total sample of 206 CTG records.

### Collecting Data on Sharing of Supporting Documents

CTG has a section (called Study Documents) that allows investigators to share protocols, informed consent forms, and statistical analysis plans [24]. Using the information from this section, data were collected on whether CTG records provided links to these supporting documents.

### Collecting Data on Sharing of IPD

CTG provides a section (called Individual Participant Data (IPD) Sharing Statement, hereafter called IPD Sharing Statement) in which investigators may divulge their plans for sharing IPD with other researchers [24]. Within the IPD Sharing Statement, we examined the following subsections: Plan to Share IPD, Plan Description, Access Criteria, and Time Frame. Data were collected from each of these subsections for records' intentions to share and, when applicable, how they shared or intended to share IPD. More specifically, data were collected on:

1. Whether the investigators stated they planned to share their data in the Plan to Share IPD subsection.
2. Whether the investigators stated they plan to share their data with unaffiliated researchers in either the Plan Description or the Access Criteria subsections. Note that plans that specified they only intended to share summary data or genetic sequencing data were categorized as a “no.” Statements that IPD would only be shared within the investigating team/affiliated institution(s) were also categorized as a “no.”
3. Whether there were any inconsistencies between the Plan to Share IPD and Plan Description/Access Criteria subsections.
4. What the timeline (if any) was for sharing data listed in the Time Frame subsection. This included both stipulation and timeframe information. In the context of this study, “stipulation” refers to any conditions where a specific activity must be completed before datasets are made available (e.g., after publication). “Timeframe” refers to any specific time or date range for sharing the datasets (e.g., within three months).
5. What the mechanism was for unaffiliated researchers getting access to the IPD listed in either the Plan Description or the Access Criteria subsections.
6. In cases where investigator contact was listed as the mechanism for getting access to IPD, whether an email address was provided anywhere in the CTG record.
7. In cases where a data sharing platform was listed as the mechanism for getting access to IPD, whether the name of the data sharing platform in which the investigators plan to share their IPD was listed in either the Plan Description or the Access Criteria subsections.
8. In cases where a data-sharing platform was listed as the mechanism for getting access to IPD, and where the platform was named, the discoverability of the study's associated dataset record in the platform. Note that for studies that named a specific data-sharing platform but didn't provide a direct link to the dataset record, the platform was searched using the study's NCT number (i.e., unique identifiers assigned to clinical studies registered in CTG [26]) or, if the latter retrieved no results, using the study's title from its corresponding CTG record.
9. For CTG records where an associated dataset record was found in a data sharing platform, whether there was a link from the dataset record back to the CTG record.

Notes that informed data entry (including quotations from the Plan Description and Access Criteria subsections) were also included. Any questionable items (i.e., where the primary investigator (PI) was uncertain of data points due to ambiguous language in the study record) were referred to the PI's coauthor and resolved via consensus.

### Collecting Data on Sharing of Publications

Publications can be linked in CTG records in two different ways:

1. **Manual links:** In the More Information section of CTG records, study investigators can manually provide links to publication records in PubMed (for the purpose of this study, “publication record” will hereafter refer to publication records in PubMed) [23]. Investigators have the option of labelling these manual links as either results or reference publications, with results publications referring to publications that report on the results of the study, and reference publications referring to works the study is citing.
2. **Automatic links:** Automatic links to publication records are automatically added to the More Information section of the CTG record. These links are generated if the NCT number of the study was included in the publication record [27–30]. Unlike manual links, automatic links do not have labels to distinguish between results or reference publications.

Publication records can link *back* to CTG records in three different ways:

3. **Associated Data links:** Associated Data links, which go directly to the CTG record, are added by publishers and/or staff at the National Library of Medicine to the publication record [31].
4. **Abstract links:** Abstract links are links to the CTG record that are within the text of the publication record's abstract. These links go directly to the CTG record.
5. **LinkOut links:** LinkOut links are automatically assigned to publication records whenever a link to the publication record is added to a CTG record within CTG. LinkOut links are indirect, meaning that when a user clicks one, the user will be taken to a search of the publication record's PubMed ID (PMID) in CTG [29].

With this information in mind, data were collected on the following:

1. The link to the publication record listed in the CTG record (i.e., the link from the record was copy-pasted into the Excel sheet).
2. Whether the linked publication record was an automatic link.
3. For publication record links added manually to the CTG record, their categorization (i.e., as a “results_reference” or “reference”) when using the XML view in the CTG record. Note that the XML view is accessible by adding “?resultsxml=true” to the end of the CTG record link [25, 29, 30].
4. Whether the publication record could be retrieved when searching the NCT number with the [si] field tag in PubMed. Note that the [si], or Secondary Source ID, field contains information relating to a variety of data, including any available NCT numbers associated with a publication [29, 32]. NCT numbers are automatically added to the [si] field when PubMed's algorithm finds NCT numbers in the abstract of a publication record [25].
5. Whether the publication record could be retrieved when searching the NCT number with the [tw] field tag in PubMed. Note that, according to the PubMed User Guide, the [tw], or Text Words, field includes “all words and numbers in the title, abstract, other abstract, MeSH terms, MeSH subheadings, publication types, substance names, personal name as subject, corporate author, secondary source, comment/correction notes, and other terms in the PubMed record” [33].
6. The PMID of the publication record. 7. Whether the publication record had an Associated Data link back to the CTG record.
7. Whether the publication record had a LinkOut link back to the CTG record.
8. Whether the publication record had an abstract link back to the CTG record.

Publications were also evaluated for whether they could be categorized as full results publications for their associated CTG record (i.e., full, original research publications reporting on the results of the study, and that study alone). To do this, a decision tree was created (to access the decision tree, see Appendix C). Any questionable items were referred to the PI's coauthor and were resolved via consensus.

## RESULTS

**Figure 1:**
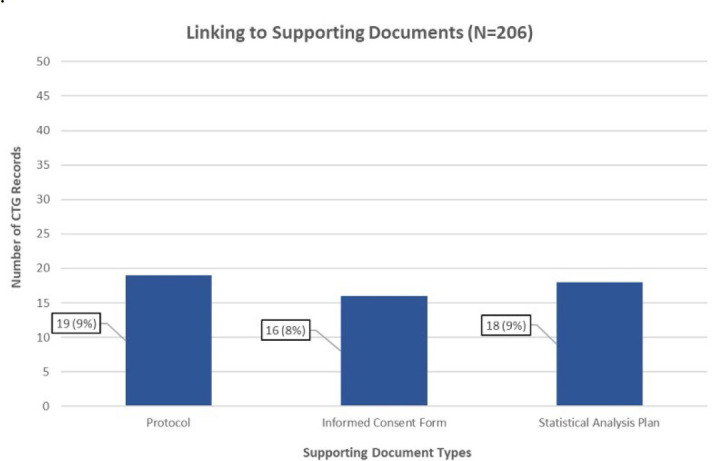
Number of CTG records that linked to protocols, informed consent forms, or statistical analysis plans

Records were exported from CTG on June 6, 2022. 206 CTG records were included in the analysis. See Appendix D: Table 1 and Figure 1 for information on the CTG records' characteristics.

**Table 1:** Intentions to share and provision of information to access IPD in the CTG records.

**A. Intentions to share IPD in Plan to Share IPD**				
**Response**	**All Records (N=206)**		**Completed Records (N=42)**	
Yes	53 (26%)		12 (29%)	
No	62 (30%)		14 (33%)	
Undecided	22 (11%)		1 (2%)	
Irrelevant^*^	69 (33%)		15 (36%)	
**B. Intentions to share IPD in Plan Description/Access Criteria**				
**Response**	**All Records (N=206)**		**Completed Records (N=42)**	
Yes	48 (23%)		12 (29%)	
No	17 (8%)		1 (2%)	
Undecided	10 (5%)		1 (2%)	
Irrelevant^*^	131 (64%		28 (67%)	
**C. Inconsistencies between A and B^†^**				
**Response**	**All Records (N=206)**	**Subset of All Records (N=10)**	**Completed Records (N=42)**	**Subset of Completed Records (N/A)**
Yes, No	7 (3%)	7 (70%)	0 (0%)	N/A
No, Yes	1 (0.5%)	1 (10%)	0 (0%)	N/A
No, Undecided	1 (0.5%)	1 (10%)	0 (0%)	N/A
Undecided, Yes	1 (0.5%)	1 (10%)	0 (0%)	N/A
**D. Mechanisms for accessing IPD in Plan Description/Access Criteria^§^**				
**Response**	**All Records (N=206)**	**Subset of All Records (N=55)**	**Completed records (N=42)**	**Subset of Completed Records (N=12)**
Upon request (i.e., via email)	14 (7%)	14 (25%)	2 (5%)	2 (17%)
Data sharing platform	18 (9%)	18 (33%)	8 (19%)	8 (67%)
Unspecified^¶^	23 (11%)	23 (42%)	2 (5%)	2 (17%)
**E. Access information for contacting investigators anywhere in CTG record (only for records stating they planned to share IPD upon request, i.e., via email)** ^§^				
**Response**	**All records (N=206)**	**Subset of All Records (N=14)**	**Completed records (N=42)**	**Subset of Completed records (N=2)**
Email address provided of at least 1 investigator	9 (4%)	9 (64%)	1 (2%)	1 (50%)
Email address not provided	5 (2%)	5 (36%)	1 (2%)	1 (50%)
**F. Access information for data sharing platforms in Plan Description/Access Criteria (only for records stating they planned to share IPD via a data sharing platform)** ^§^				
**Response**	**All records (N=206)**	**Subset of All Records (N=18)**	**Completed records (N=42)**	**Subset of Completed records (N=8)**
Platform named	16 (8%)	16 (89%)	6 (14%)	6 (75%)
Platform not named	2 (1%)	2 (11%)	2 (5%)	2 (25%)

* In (A), “Irrelevant” indicates that there was no IPD Sharing Statement section, (and therefore no Plan to Share IPD subsection). For (B), it indicates there was no Plan Description nor Access Criteria subsections in the record.

† In (C), the responses are organized by the Plan to Share IPD followed by the Plan Description/Access Criteria responses. For example, “Yes, No” indicates the records stated “Yes” in their Plan to Share IPD subsection, but “No” in their Plan Description/Access Criteria subsections.

§ (C), (D), (E), and (F) include the total number of records followed by a specific subset. For (C) the subset (10, or 0 for completed) is the number of CTG records that had inconsistencies between (A) and (B). For (D) the subset (55, or 12 for completed) is the number of records that stated “Yes” in either (A) or (B). For (E) the subset (14, or 2 for completed) is the number of records that listed “Upon request” (i.e., via email) in (D). For (F) the subset (18, or 8 for completed) is the number of records that listed “Data sharing platform” in (D).

¶ In (D), “Unspecified” indicates no mechanism for unaffiliated researchers accessing IPD was indicated in the Plan Description nor Access Criteria subsections in the record.

### Sharing of Supporting Documents

Of the 206 CTG records, 19 (9%) provided links to protocols, 16 (8%) to informed consent forms, and 18 (9%) to statistical analysis plans (see Figure 1). Only 8 (4%) CTG records contained links to all 3 supporting document types.

All supporting document links were functional and allowed users to access documents as downloadable PDFs.

### Sharing of IPD

Of the 206 CTG records, 53 (26%) stated “Yes” (12 or 29% of the 42 records marked as completed) in their Plan to Share IPD subsection for their intentions to share IPD. 69 (33%) did not have a Plan to Share IPD Statement (15 or 36% for completed) (see A in Table 1). In their Plan Description/Access Criteria subsections, 48 (23%) of the 206 records indicated “Yes” (12 or 29% of the 42 completed records) for their intention to share IPD. 131 (64%) of the 206 records (28 or 67% of the 42 completed) did not have a Plan Description nor Access Criteria subsection (see B in Table 2). Inconsistencies were identified in 10 (5%) of the 206 CTG records when comparing their Plan to Share IPD subsection with their Plan Description/Access Criteria subsections for their intentions to share IPD. No completed records had inconsistencies (see C in Table 1).

**Table 2:** Stipulations and timeframes the CTG records listed for sharing IPD.

**All records that stated they intended to share IPD (N=55)**				
**Stipulation, with or without timeframe**	**Records**	**Range**	**Mean**	**Median**
No stipulation, no timeframe	14 (25%)	N/A	N/A	N/A
After publication, with timeframe	11 (20%)	0 to 36 months	10 months	9 months
No stipulation, with timeframe	7 (13%)	0 to 9 months	3 months	0 months
After study completion, with timeframe	6 (11%)	6 to 84 months	22 months	11 months
After study completion, no timeframe	5 (9%)	N/A	N/A	N/A
No stipulation, year named	5 (9%)	2021 to 2026	2023	2023
After publication, no timeframe	3 (5%)	N/A	N/A	N/A
After database lock, with timeframe	3 (5%)	24 months	24 months	24 months
After “first survey collected,” with timeframe	1 (2%)	6 months	6 months	6 months
**Completed records that stated they intended to share IPD (N=12)**				
**Stipulation, with or without timeframe**	**Records**	**Timeframe**	**Mean**	**Median**
No stipulation, no timeframe	3 (25%)	N/A	N/A	N/A
After publication, with timeframe	4 (33%)	0 to 12 months	7.5 months	9 months
No stipulation, with timeframe	0 (0%)	N/A	N/A	N/A
After study completion, with timeframe	2 (17%)	6 to 84 months	45 months	45 months
After study completion, no timeframe	1 (8%)	N/A	N/A	N/A
No stipulation, year named	1 (8%)	2021	2021	2021
After publication, no timeframe	0 (0%)	N/A	N/A	N/A
After database lock, with timeframe	1 (8%)	24 months	24 months	24 months
After “first survey collected,” with timeframe	0 (0%)	N/A	N/A	N/A

Of the 55 records that stated they intended to share IPD in either their Plan to Share IPD or their Plan Description/Access Criteria subsections (this number including records that had inconsistencies with at least one response being “Yes”), 32 (58%) (16% of all 206 records) indicated a mechanism for how they would share IPD, being either upon request or via a data sharing platform. When limiting to the 12 records with a completed status that stated they intended to share IPD, 10 (83%) (24% of all 42 completed records) indicated a mechanism for how they would share IPD (see D in Table 1).

Of the 14 records that intended to share IPD upon request (i.e., via email), 9 (64%) (4% of all 206 records) provided an email address for at least 1 investigator somewhere in the CTG record. When limiting to the 2 completed records that intended to share IPD upon request, 1 (2% of all 42 completed records) provided an email address (see E in Table 1).

Of the 18 records that stated they intended to share via a data sharing platform, 16 (89%) (8% of all 206 records) named a specific data sharing platform. When limiting to the 8 completed records that stated they intended to share via a data sharing platform, 6 (75%) (14% of all 42 completed records) named a specific data sharing platform (see F in Table 1). None of the records, regardless of status, linked to a dataset record in a data sharing platform; only 1 dataset record (being from a CTG record with a completed status) was found by searching the named data sharing platform, which did link back to its corresponding CTG record.

**Figure 2:**
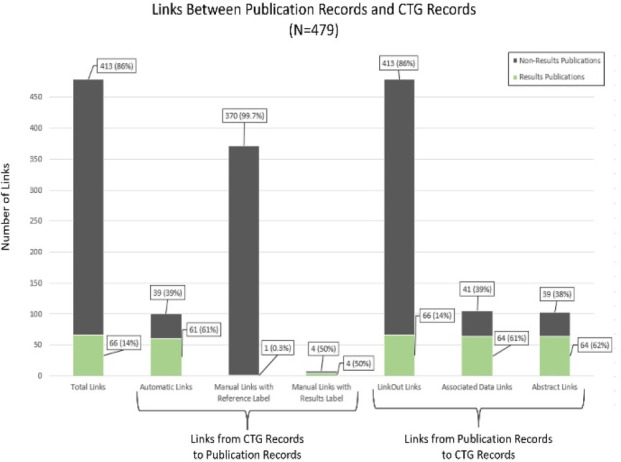
Number and types of links between publication records (including non-results and results publication records) and their corresponding CTG records.

**Figure 3:**
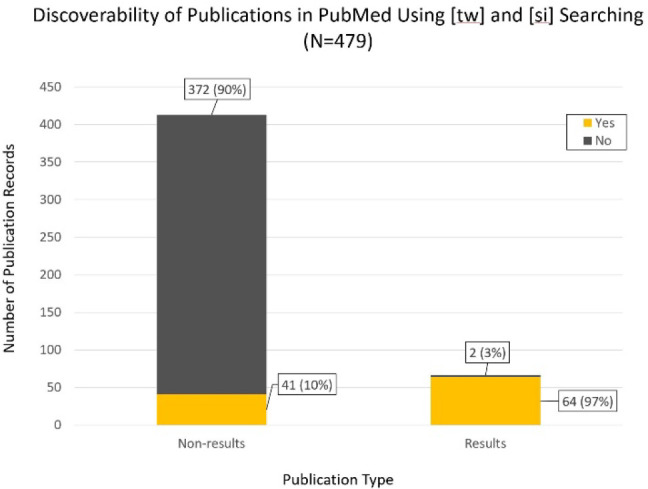
Number of non-results and results publications retrieved/not retrieved by searching the NCT number using [tw] and [si] field tags in PubMed.

Cumulatively, 25 (45% of the 55 CTG records that stated they intended to share IPD or 12% of all 206 CTG records) provided sufficient information to access IPD (i.e., they provided either an email address for records that stated they would share IPD via email, or named a data sharing platform for those that stated they would share IPD via a data sharing platform). When limiting to records with a completed status, 7 records (58% of those that stated they intended to share IPD, or 17% of all 42 completed records) provided sufficient information to request access to IPD (see E and F in Table 1. For a breakdown of this data by record start year, see Appendix D: Figures 2 and 3).

Stipulations and timeframes for sharing IPD were variable. Of the 55 records stating they intended to share IPD, 22 (40%) did not share a specific timeframe for sharing their IPD. When limiting to the 12 records with a completed status that stated they would share IPD, the number was 4 or 33%. The most common stipulation was that IPD would be shared after the investigators published their findings in a journal with a specified timeframe (11 or 20% of the 55 CTG records, 4 or 33% of the 12 completed records), with the specified timeframe after publication being anywhere between immediate and 36 months (or immediate to 12 months for the completed records) (see Table 2).

### Sharing of Publications

Of the 206 CTG records, 80 (39%) provided one or more links to publication records. Of the 42 completed records, 26 (62%) provided one or more links to publication records. 43 (21%) of the 206 CTG records, including 18 or 43% of completed records, linked to at least 1 results publication (see Table 3. For a breakdown of these data by record start year, see Appendix D: Figures 4 and 5).

**Table 3:** Number and proportion of CTG records that had links to publication records, and summative statistics on number of links in the CTG records.

**Records with X number of links**	**All records (N=206 records)**	**Completed records (N=42 records)**
Records with 0 links (any type)	126 (61%)	16 (38%)
Records with only 1 link (any type)	36 (17%)	13 (31%)
Records with > 1 < 10 links (any type)	35 (17%)	11 (26%)
Records with > 10 links (¿my type)	9 (4%)	2 (5%)
Records that *only* linked to non-results publications	37 (18%)	8 (19%)
Records that linked to at least 1 results publication	43 (21%)	18 (43%)
**Summative statistics for number of links in CTG records**	**Links in all records (N=479 links)**	**Links in completed records (N=114 links)**
Mean number of links	2.33 (SD=7.77)	2.71 (SD=7.87)
Median number of links	0	1
Maximum number of links	52	50
Minimum number of links	0	0
**Summative statistics for number of results links in CTG records**	**Results links in all records (N=66 links)**	**Results links in completed records (N=20 links)**
Mean number of links	0.32 (SD=0.87)	0.48 (SD=0.63)
Median number of links	0	0
Maximum number of links	6	3
Minimum number of links	0	0

There were 479 total links to publication records in the sample of 206 CTG records, with 66 (14%) of these being links to results publication records (see Figure 2).

For links from the CTG records to the publication records, 100 were automatic links, with 61 being links to results publication records. 371 were manual links and were assigned a “Reference” label, with just 1 of these linking to a results publication record. 8 manual links were assigned a “Results” label, with half of these linking to a results publication record (see Figure 2).

For links from publication records back to their corresponding CTG records, all 479 publication records linked back to their CTG records using a LinkOut link. 105 publication records linked back to their CTG record using an Associated Data Link, with 64 (61%) of these being results publication records. 103 publication records linked to their CTG records using an Abstract Link, with 64 (62%) of these being results publication records (see Figure 2).

Sixty-four (97%) of the 66 linked results publication records could be found in PubMed by searching NCT number using the [si] or [tw] field tags. Retrieval of linked non-results publications was significantly less, with 41 (10%) of the 413 records being retrieved (see Figure 3).

## DISCUSSION

### Sharing of Supporting Documents

Accessibility of supporting documents increases the transparency and utility of clinical study results [6, 34]. The submission of protocols and statistical analysis plans to CTG is a requirement for all clinical trials receiving NIH funding. This requirement has been in place since 2017, with an extension to 2024 for basic experimental studies involving human participants. The submission of protocols and statistical analysis plans is additionally required by studies subject to the FDA's Final Rule for Clinical Trials Registration and Results Information Submission since 2017 [17, 18].

As indicated by this study, the sharing of supporting documents in federally funded COVID-19 CTG records is uncommon, with only 4% of CTG records in this study sharing all three supporting document types. A few studies have touched on the availability of supporting documents in clinical studies, including Gaba et al (who reported 11% for sharing protocols and 9% statistical analysis plans in a subset of non-commercially funded clinical trial CTG records), and Kapp et al (who reported a higher 38% for the sharing of protocols and 29% for statistical analysis plans for a subset of COVID-19 trial publications) [35, 6]; however, there is a dearth of studies that have examined the availability of these documents in the CTG record, itself, especially within the context of COVID-19. More studies are needed to evaluate the availability of these documents in these CTG records, and there needs to be greater effort to encourage investigators to link these documents in CTG records. Additionally, separate attention should be paid to the format in which supporting documents are shared, as the addition of computable formats, such as HTML, could facilitate more in-depth analyses.

### Sharing of IPD

Sharing IPD deriving from clinical studies increases the validity, transparency, reproducibility, and utility of study results; facilitates the ability for unaffiliated researchers to build upon past discoveries; reduces study redundancy; and assists in fulfilling the ethical obligation to study participants of maximizing the impact of study findings [2, 8–12]. Though not required, IPD sharing is strongly encouraged by the NIH and the ICMJE, with both requiring the submission of data management and sharing plans for studies funded by the NIH (as of January 25, 2023) or published by ICMJE membership journals (as of January 2019) [8,10,12,15,36].

As indicated by this study, intentions to share IPD for federally funded COVID-19 studies in CTG can be improved. The study found that only 27% of the 206 records (or 29% of 42 records with a completed status) indicated they planned to share their IPD with unaffiliated researchers. This number, while suboptimal, was slightly greater than estimates from previous studies, with Li et al. and Larson et al. both being at around 15% for subsets of CTG records for COVID-19 studies [8,9]; and Begeris et al, Ohmann et al., and Gaba et al. being between 10% and 12% for subsets of clinical trial CTG records (not limited to COVID-19) [34,35,37]. These consistently low numbers, both in this study and in these past studies, are likely reflective of the reservations that investigators have towards sharing data, which include fears of privacy risks to study participants, unaffiliated researchers misusing or misinterpreting data, and lack of proper attribution [38]. However, in terms of fearing privacy risk to participants, it is important to note that clinical study participants themselves are supportive of data sharing, even when considering potential risks to themselves as a result of this sharing [11]. With regard to proper attribution, research has shown that studies that share data tend to be cited more frequently than those that don't [39, 40]. There should be more effort on the part of organizations and librarians to inform investigators of these ethical and professional benefits from sharing research data. Librarians can also provide resources on where and how to share data, including available clinical data sharing platforms for depositing data and where to find guidance on how to safely share IPD, such as that provided by the Inter-university Consortium for Political and Social Research [41].

An additional area that could be improved is the availability and standardization of IPD sharing plans in CTG. Of the 206 records, a surprising 33% (or 36% of the 42 completed records) had no IPD Sharing Statement in the CTG record, being a finding that reinforces that of Gaba et al., who found that 23% of a subset of noncommercially funded clinical trial CTG records didn't have an IPD sharing plan [35]. Additionally, a few records had discrepancies between their IPD Sharing Plan and their Plan Description/Access Criteria subsections, an observation that was similarly remarked upon by Larson et al. and Bergeris et al., who also noted instances of these discrepancies in the subsets of clinical trial CTG records they examined [9, 37]. Listed mechanisms for IPD sharing were also lacking, with only 58% of the 55 records that indicated they intended to share IPD (or 16% of all 206 records) providing information on the mechanism by which they would share IPD (as a note, this number did increase slightly when limiting to the 12 completed records that stated they intended to share IPD, with 83% of the 12 records, or 24% of all 42 completed records, providing a mechanism by which they would share IPD); though this finding was an improvement compared to Larson et al and Gaba et al., which had been 26.6% and 6%, respectively [9, 35]. However, the number was lower when further limiting to studies that provided both a mechanism and an email address or the name of the data sharing platform, when applicable, which was only 45% of the 55 records (58% of the 12 completed records), or just 12% of all 206 CTG records (17% of all 42 completed records). Finally, stipulations and timelines for sharing IPD were frequently ambiguous and lacked standardization. The complete exclusion of IPD plans, and the discrepancies, lack of information for requesting access to IPD, and unstandardized data sharing timelines reflect the need for increased guidance, transparency, and standardization, a need particularly vital with the current NIH and ICMJE requirements for submissions of IPD sharing plans [12, 36]. Librarians can assist in this area by providing workshops and guidance for how to create and what information to include in data sharing plans, including how to make data more discoverable. CTG, as a platform, may also consider developing automated machine learning approaches to parsing the data sharing plan and related structured fields in the CTG record and providing feedback to study record administrators during the record review stage. Such a review step is already in place for some parts of the CTG record and extending it with additional review of the IPD sharing plan would require minimal process changes. This addition could assist in alerting administrators to missing or discrepant information in IPD sharing plans and increase standardization of these plans.

### Sharing of Publications

Scholarly publications deriving from clinical studies support evidence-based decision making and, especially in the context of pandemics, serve as an invaluable vehicle for disseminating knowledge to control and manage disease [13, 14]. Following the encouragement of the National Science and Technology Advisors and Wellcome Trust, among others, a number of publishers volunteered to make publications relating to COVID-19 research publicly available during the pandemic [19–21]. Additionally, the Nelson Memo calls for the public availability of publications deriving from federally funded research, among other research materials [22].

As demonstrated by this study, links from federally funded COVID-19 CTG records to publications (and vice versa) could be improved. In terms of links from CTG records to publication records, only 21% of the 206 CTG records (43% of the 42 completed records) linked out to at least 1 results publication, being slightly higher than Huser & Mayer in their study of COVID-19 clinical trials, which had been at 17.8% for all COVID-19 trials, regardless of funding source [25]. To be fair, the low number of linked results publications may be attributed to the relative recentness of the COVID-19 pandemic, the investigating teams conducting these studies not having had sufficient time to publish their results in a journal.

Of note, the vast majority (86%) of linked publication records in CTG records were not results publications. While linking to non-results publication records can give researchers related information on the clinical study, CTG records could be improved by clarifying the identity of these linked records, being a view that isn't unique to this study [27, 29]. Though this distinction can be accessed in the XML view of CTG for manually linked publications, it is not available for automatically linked publications. The absence of these labels complicates the detection of automatically linked results publications within a CTG record, as they are frequently interspersed with automatic links to non-result publications. Because so many results publications (92%) in this study were linked automatically, it would be beneficial to introduce results and non-results labelling to automatically linked publication records. One potential solution could be having CTG notify investigators when a publication has been detected by the algorithm that assigns these automatic links and require investigators to confirm whether the publication is a results publication prior to it being added to the CTG record.

The extent to which publication records linked back to CTG records was promising, with the majority (97%) of the 66 results publications linking back to their corresponding CTG record using an Associated Data link and/or abstract link. Associated Data and abstract links are arguably preferred over the LinkOut links, as the former two link directly to the CTG record rather than the results of a PMID search in CTG, as is the case with LinkOut links. Unlike LinkOut links, they also include the NCT number in the links, which can be retrieved by an [si] search (in the case of Associated Data links) or [tw] search in PubMed. However, all the link types could benefit from better labelling, as there is currently no quick way of distinguishing results from non-result publication records in PubMed. While ICMJE requires the provision of registration numbers (e.g., NCTs) in the abstracts of clinical trial publications [42], they do not require specific language acknowledging that the publication is reporting upon the results of the trial in the abstract. This complicates the detection of results publications, as numerous non-results publications in the sample also included NCT numbers in their abstracts. One way to mediate this issue would be for librarians and other stakeholders such as ICMJE to encourage investigators to include specific statements in the abstract that the publication is reporting on the results of the study. Consistent and standardized usage of language such as this could also pave the way for future, automatic labelling of results publications in PubMed.

Promisingly, searching by NCT number using [si] and [tw] searching in PubMed was effective in retrieving results publication records linked in CTG. The efficacy of [si] and [tw] searching is not altogether surprising, as the inclusion of NCT numbers in the abstracts of results publications is required by ICMJE [42]. Better labelling, however, could be applied to the metadata of PubMed records to better identify results publications, as both [tw] and [si] searching retrieved results as well as non-results publications.

### Limitations and Conclusion

There were limitations to this study. Due to time constraints, only studies containing at least 200 participants were included in the analysis. It's possible that the recency of the COVID-19 pandemic may have also affected the results of this study (i.e., that, given more time, research teams may have shared more research materials in CTG), especially as the majority (80%) didn't have a completed status. Even so, during pandemics the rapid dissemination of research materials is critical, and this study provides valuable insight into the current state of these sharing intentions and practices.

As indicated by this study, improvements can be made to the discoverability of research materials in CTG records for federally funded COVID-19 studies. Sharing these materials on CTG can increase the discoverability of these materials, and therefore contribute to increasing the validity, transparency, and reusability of clinical research.

## Data Availability

All data and data dictionaries associated with this study can be accessed at the study Github repository at: https://github.com/weepai/Discoverability-of-supporting-research-materials-for-U.S.-federally-funded-Covid-19-clinical-studies.
