# Supplementary material for: Evaluating the discoverability of supporting research materials in ClinicalTrials.gov for US federally funded COVID-19 clinical studies
Source: J Med Libr Assoc. 2024 Jul 29;112(3):250–60. doi: 10.5195/jmla.2024.1799 (PMC11412123; doi:10.5195/jmla.2024.1799)
Supplement: Supplementary file 1 — Appendix A [file jmla-112-3-250-s01.docx]

**Appendix A: Glossary**

Contents

[Abstract links 2](#_Toc147915293)

[Associated Data links 2](#_Toc147915294)

[Automatic links 3](#_Toc147915295)

[CTG 3](#_Toc147915296)

[CTG Record 3](#_Toc147915297)

[Investigators 3](#_Toc147915298)

[IPD 3](#_Toc147915299)

[IPD Sharing Statement (includes Plan to Share IPD, Plan Description, Access Criteria, and Time Frame Subsections) 3](#_Toc147915300)

[LinkOut links 4](#_Toc147915301)

[Manual links 4](#_Toc147915302)

[NCT Number 5](#_Toc147915303)

[Publication Record 5](#_Toc147915304)

[Non-results Publication 5](#_Toc147915305)

[PMID 5](#_Toc147915306)

[Results Publication 5](#_Toc147915307)

[Stipulation (Timeline) 5](#_Toc147915308)

[Timeframe (Timeline) 6](#_Toc147915309)

[Unaffiliated Researcher 6](#_Toc147915310)

[[si] 6](#_Toc147915311)

[Supporting Documents 6](#_Toc147915312)

[[tw] 6](#_Toc147915313)

# Abstract links

Abstract links are links to the ClinicalTrials.gov record that are within the text of the publication record's abstract in PubMed. These links go directly to the ClinicalTrials.gov record. They typically include an NCT number (i.e., a unique identifier assigned to studies registered in ClinicalTrials.gov) within the link, itself (meaning they can be searched by NCT number in PubMed).

Below is an example of an abstract link in a publication record in PubMed.


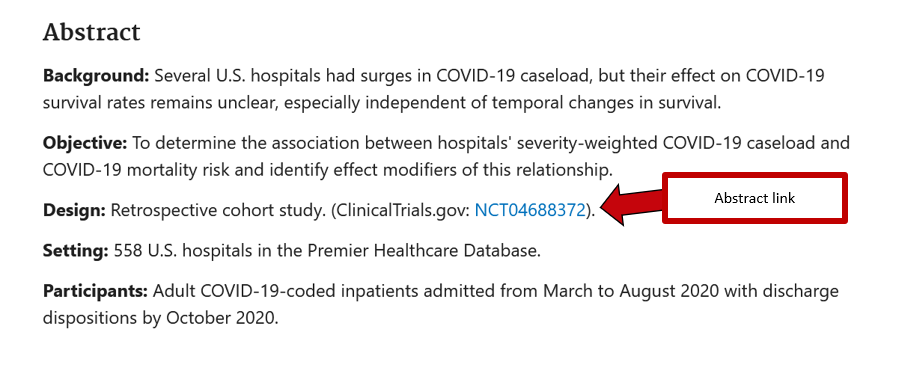


# Associated Data links

Associated Data links, which go directly to the ClinicalTrials.gov record, are added by publishers and/or staff at the National Library of Medicine to the publication record in PubMed [1]. They typically include an NCT number (i.e., a unique identifier assigned to studies registered in ClinicalTrials.gov) within the link, itself (meaning they can be searched by NCT number in PubMed).

Below is an example of an Associated Data link in a publication record in PubMed (they are typically at the very bottom of the record):


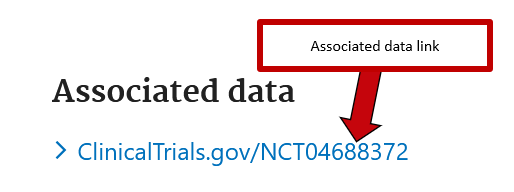


# Automatic links

Automatic links to publication records are automatically added to the More Information section of the ClinicalTrials.gov record. These links are generated if the NCT number (i.e., unique identifiers assigned to clinical studies registered in ClinicalTrials.gov [2]) of the study was included in the publication record in PubMed [3-6].

Below is an example of an automatically linked publication in ClinicalTrials.gov record.


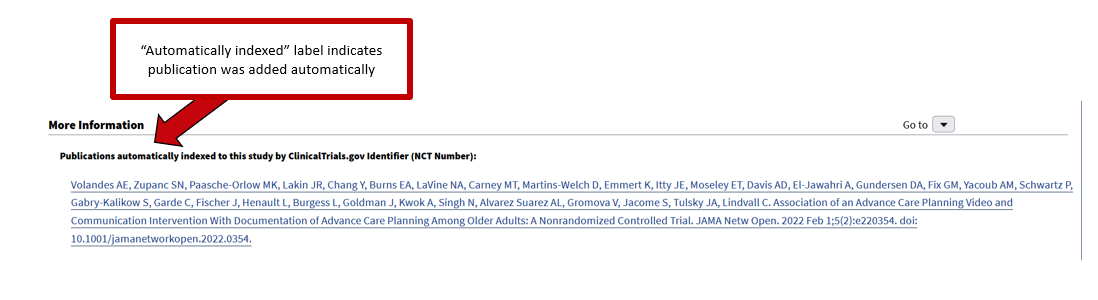


# CTG

Acronym for ClinicalTrials.gov used by the authors of the manuscript.

# CTG Record

Short for "ClinicalTrials.gov record." These are clinical studies registered in ClinicalTrials.gov.

# Investigators

Within the context of this study, the researchers that conducted the clinical study.

# IPD

Acronym for Individual Participant Data used by the authors of the manuscript.

# IPD Sharing Statement (includes Plan to Share IPD, Plan Description, Access Criteria, and Time Frame Subsections)

Short for "Individual Participant Data (IPD) Sharing Statement." Section of a ClinicalTrials.gov record in which investigators may divulge their plans for sharing IPD with other researchers [7]. IPD Sharing Statements consist of subsections, including Plan to Share IPD, Plan Description, Access Criteria, and Time Frame (among a few others). Below is a screenshot of what an IPD Sharing Statement might look like in a ClinicalTrials.gov record.


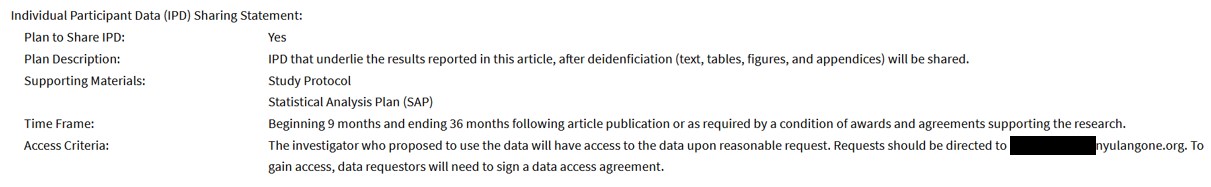


# LinkOut links

LinkOut links are automatically assigned to publication records whenever a link to a publication record from PubMed is added to a ClinicalTrials.gov record within ClinicalTrials.gov. These links are indirect, meaning that, when a user clicks one, the user will be taken to a search of the publication record's PubMed ID (PMID) in ClinicalTrials.gov [5]. LinkOut links do not have NCT numbers (i.e., unique identifiers assigned to records in ClinicalTrials.gov) within the link, itself (meaning they aren't searchable in PubMed by NCT number).

Below is an example of a LinkOut link in a publication record in PubMed (they are typically at the very bottom of the record):


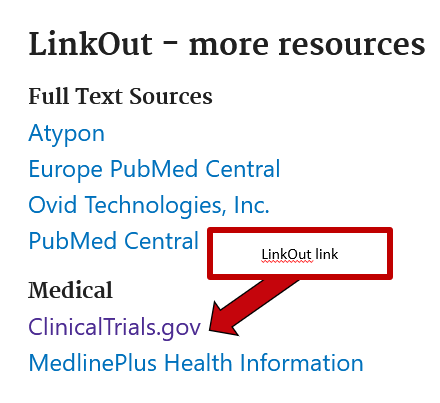


# Manual links

In the More Information section of ClinicalTrials.gov records, study investigators can manually provide links that go to publication records in PubMed [8]. Investigators have the option of labelling these manual links as either results or reference publications, with results publications referring to publications that report on the results of the study, and reference publications referring to works the study is citing.

Below is a screenshot of what a manual link looks like in ClinicalTrials.gov.


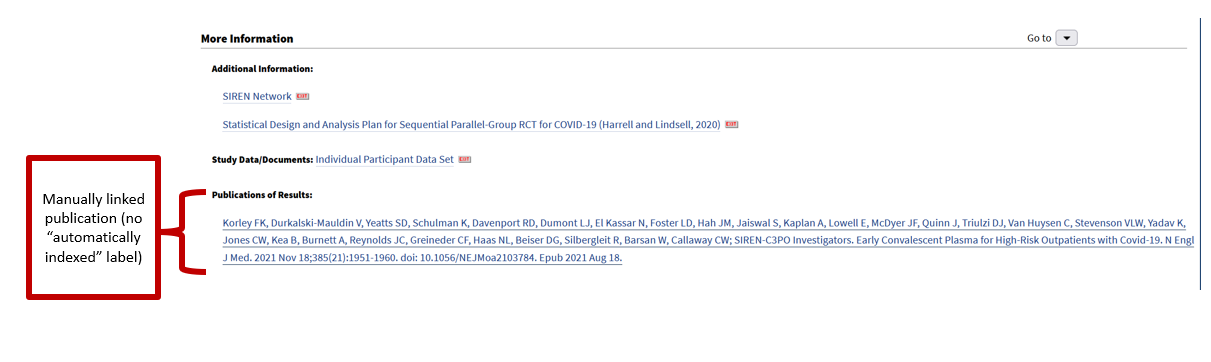


Below is a screenshot of what a manual link looks like in ClinicalTrials.gov using the XML view when it has been assigned a "Results" label.


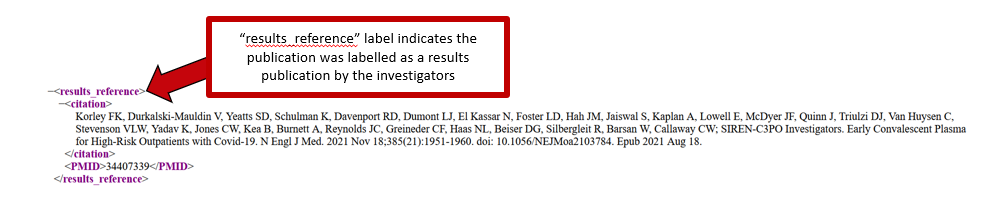


And below is a screenshot of what a manual link looks like in ClinicalTrials.gov using the XML view when it has been assigned a "Reference" label


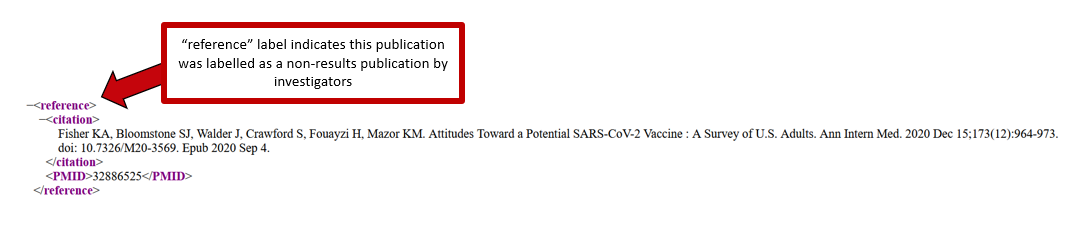


# NCT Number

Unique identifiers assigned to clinical studies registered in ClinicalTrials.gov [2]

Publication Record

In the context of this study, publication records refer to publication records in the PubMed database.

# Non-results Publication

In the context of this manuscript, publications that are *not* full, original research publications reporting on the results of the study, and that study alone.

# PMID

PubMed identifier. These are assigned to records indexed in PubMed.

# Results Publication

In the context of this manuscript, a full, original research publication reporting on the results of the study, and that study alone.

# Stipulation (Timeline)

In the context of this manuscript, any conditions where a specific activity must be completed before datasets are made available (e.g., after publication). This information is listed in the "Time Frame" subsection of a ClinicalTrials.gov record.

# Timeframe (Timeline)

In the context of this manuscript, any specific time or date range for sharing the datasets (e.g., within 3 months). This information is listed in the "Time Frame" subsection of a ClinicalTrials.gov record.

# Unaffiliated Researcher

In the context of this manuscript, researchers not affiliated with the clinical study in question.

# [si]

PubMed field tag. The [si], or Secondary Source ID, field contains information relating to a variety of data, including NCT numbers (i.e., unique identifiers for studies registered in ClinicalTrials.gov) associated with a publication, if available [5, 9]. NCT numbers are automatically added to the [si] field when PubMed's algorithm finds NCT numbers in the abstract of a publication record in PubMed [10].

# Supporting Documents

In this manuscript, supporting documents refer to protocols, statistical analysis plans, and informed consent forms. This information can be found in the "Study Documents" section of a ClinicalTrials.gov record (as can be seen in the screenshot below).


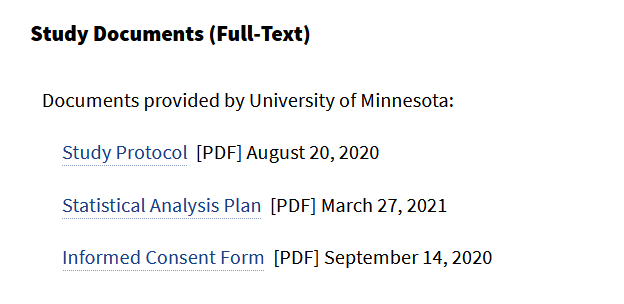


# [tw]

PubMed field tag. According to the PubMed User Guide, the [tw], or Text Words, field includes "all words and numbers in the title, abstract, other abstract, MeSH terms, MeSH subheadings, publication types, substance names, personal name as subject, corporate author, secondary source, comment/correction notes, and other terms in the PubMed record" [11].

References

1. Email from NLM Support Team. Email message to: **[redacted]**. National Library of Medicine Help Desk; 2023

2. Glossary of common site terms: ClinicalTrials.gov; 2021 [cited 2 Feb 2023]. Available from: <https://clinicaltrials.gov/ct2/about-studies/glossary>.

3. Salholz-Hillel M, Strech D, Carlisle BG. Results publications are inadequately linked to trial registrations: an automated pipeline and evaluation of German university medical centers. Clin Trials. 2022 Apr 1:337-46. DOI: 10.1177/17407745221087456.

4. Bashir R, Bourgeois FT, Dunn AG. A systematic review of the processes used to link clinical trial registrations to their published results. Syst Rev. 2017 Jul 3;6(1):1-17. DOI: 10.1186/s13643-017-0518-3.

5. Huser V, Cimino JJ. Precision and negative predictive value of links between ClinicalTrials.gov and PubMed. AMIA Annu Symp Proc. 2012;2012:400-8.

6. How to find results of studies: ClinicalTrials.gov; 2021 [cited 2 Feb 2023]. Available from: <https://clinicaltrials.gov/ct2/help/how-find/find-study-results>.

7. ClinicalTrials.gov protocol registration data element definitions for interventional and observational studies: ClinicalTrials.gov; 2020 [cited 2 Feb 2023]. Available from: https://prsinfo.clinicaltrials.gov/definitions.html.

8. ClinicalTrials.gov background: ClinicalTrials.gov; 2021 [cited 2 Feb 2023]. Available from: <https://clinicaltrials.gov/ct2/about-site/background#WhatInformationCanIFind>.

9. MEDLINE/PubMed data element (field) descriptions: National Library Of Medicine; 2019 [cited 2 Feb 2023]. Available from: <https://www.nlm.nih.gov/bsd/mms/medlineelements.html#si>.

10. Mayer CS, Huser V. regCOVID: tracking publications of registered COVID-19 studies. BMC Med Res Methodol. 2022 Aug 10;22(1):1-10. DOI: 10.1186/s12874-022-01703-9.

11. PubMed user guide: National Library of Medicine; 2023 [cited 2 Feb 2023]. Available from: <https://pubmed.ncbi.nlm.nih.gov/help/#tw>.
