# Supplementary material for: Evaluating the discoverability of supporting research materials in ClinicalTrials.gov for US federally funded COVID-19 clinical studies
Source: J Med Libr Assoc. 2024 Jul 29;112(3):250–60. doi: 10.5195/jmla.2024.1799 (PMC11412123; doi:10.5195/jmla.2024.1799)

Appendix B: Data Collection Definitions and Examples

Contents

[1. Overview 2](#_Toc147915580)

[2. Study Documents Data 2](#_Toc147915581)

[A. Protocol 2](#_Toc147915582)

[B. Protocol link functional 2](#_Toc147915583)

[C. Informed consent form 3](#_Toc147915584)

[D. Informed consent form link functional 3](#_Toc147915585)

[E. Statistical analysis plan 4](#_Toc147915586)

[F. Statistical analysis plan link functional 4](#_Toc147915587)

[3. Datasets Data 5](#_Toc147915588)

[A. Plan to share data 5](#_Toc147915589)

[B. Plan description/access criteria 5](#_Toc147915590)

[C. Plan inconsistencies 6](#_Toc147915591)

[D. Timeframe 7](#_Toc147915592)

[E. Mechanism for getting access to data 7](#_Toc147915593)

[F. Email address provided 8](#_Toc147915594)

[G. Platform name provided 9](#_Toc147915595)

[H. Discoverability 9](#_Toc147915596)

[I. Link back to CTG 10](#_Toc147915597)

[4. Publications Data 11](#_Toc147915598)

[A. Link to publication in CTG record 11](#_Toc147915599)

[B. Automatically linked 11](#_Toc147915600)

[C. Link type (for manually added publication record links) 12](#_Toc147915601)

[D. PubMed [si] test 13](#_Toc147915602)

[E. PubMed [tw] test 13](#_Toc147915603)

[F. PMID 13](#_Toc147915604)

[G. NCT number included somewhere in the publication record 14](#_Toc147915605)

[H. Associated data link back to CTG record 14](#_Toc147915606)

[I. LinkOut link back to CTG record 15](#_Toc147915607)

[J. Abstract link back to CTG record 15](#_Toc147915608)

# **1. Overview**

This document lists and defines the data elements that were included in the study, and provides examples of where such data were being collected in the records. Note that data automatically exported from ClinicalTrials.gov (hereafter referred to as CTG) is not included in this list. For additional information on the data collected, see **[GitHub link redacted for anonymity]**. For information on data collected for results verification (i.e., identifying whether a publication was a results or non-results publication) see **Appendix III.**

# **2. Study Documents Data**

## **A. Protocol**

**Is there a protocol link in the Study Documents section of the CTG record?**

- **Y: Yes** (A protocol link is present in the Study Documents section of the CTG record)
- **N: No** (No protocol is linked in the Study Documents section of the CTG record)

**Example from NCT04546581 in CTG**

Link: [https://classic.clinicaltrials.gov/ct2/show/NCT04546581](https://classic.clinicaltrials.gov/ct2/show/NCT04546581?term=NCT04546581&draw=2&rank=1)


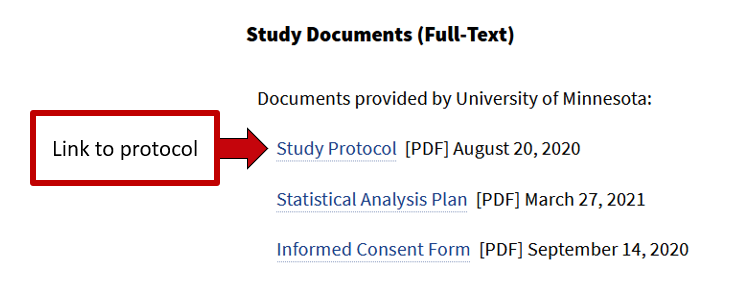


## **B. Protocol link functional**

**Is the protocol link in the CTG record functional?**

- **Y: Yes** (The protocol link in the CTG record is functional)
- **N: No** (The protocol link in the CTG record is not functional)
- **I: Irrelevant** (There is no link to a protocol in the CTG record)

**Example from NCT04546581 in CTG**

Link: [https://classic.clinicaltrials.gov/ct2/show/NCT04546581](https://classic.clinicaltrials.gov/ct2/show/NCT04546581?term=NCT04546581&draw=2&rank=1)


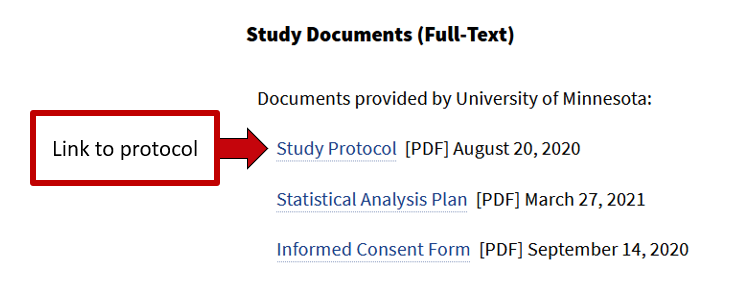


## **C. Informed consent form**

**Is there an informed consent form link in the Study Documents section of the CTG record?**

- **Y: Yes** (An informed consent form link is present in the Study Documents section of the CTG record)
- **N: No** (No informed consent form link is present in the Study Documents section of the CTG record)

**Example from NCT04546581 in CTG**

Link: [https://classic.clinicaltrials.gov/ct2/show/NCT04546581](https://classic.clinicaltrials.gov/ct2/show/NCT04546581?term=NCT04546581&draw=2&rank=1)


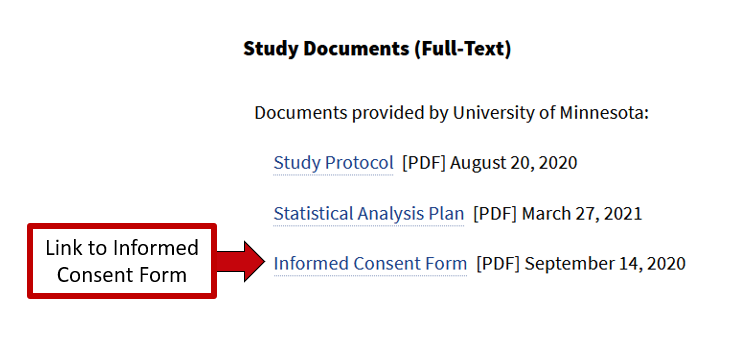


## **D. Informed consent form link functional**

**Is the informed consent form link in the CTG record functional?**

- **Y: Yes** (The informed consent form link in the CTG record is functional)
- **N: No** (The informed consent form link in the CTG record is not functional
- **I: Irrelevant** (There is no link to an informed consent form in the CTG record)

**Example from NCT04546581 in CTG**

Link: [https://classic.clinicaltrials.gov/ct2/show/NCT04546581](https://classic.clinicaltrials.gov/ct2/show/NCT04546581?term=NCT04546581&draw=2&rank=1)


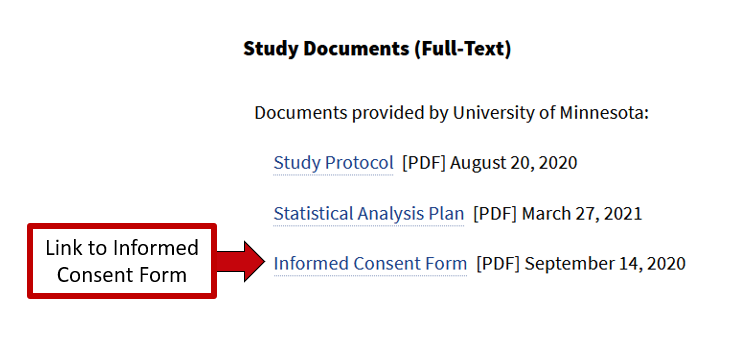


## **E. Statistical analysis plan**

**Is there statistical analysis plan link in the Study Documents section of the CTG record?**

- **Y: Yes** (A statistical analysis plan link is present in the Study Documents section of the CTG record)
- **N: No**: (No statistical analysis plan link is present in the Study Documents section of the CTG record)

**Example from NCT04546581 in CTG**

Link: [https://classic.clinicaltrials.gov/ct2/show/NCT04546581](https://classic.clinicaltrials.gov/ct2/show/NCT04546581?term=NCT04546581&draw=2&rank=1)


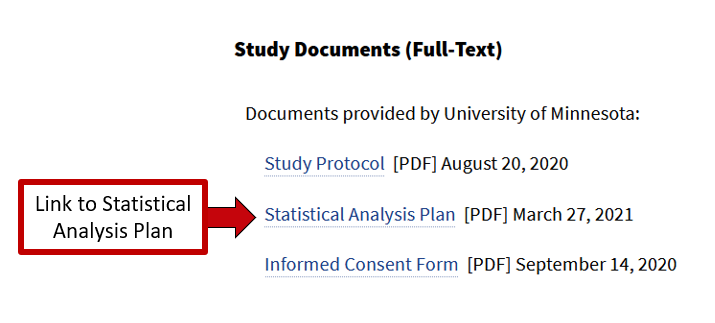


## **F. Statistical analysis plan link functional**

**Is the statistical analysis plan link in the CTG record functional?**

- **Y: Yes** (The statistical analysis plan link in the CTG record is functional)
- **N: No** (The statistical analysis plan link in the CTG record is not functional)
- **I: Irrelevant** (There is no link to a statistical analysis plan in the CTG record)

**Example from NCT04546581 in CTG**

Link: [https://classic.clinicaltrials.gov/ct2/show/NCT04546581](https://classic.clinicaltrials.gov/ct2/show/NCT04546581?term=NCT04546581&draw=2&rank=1)


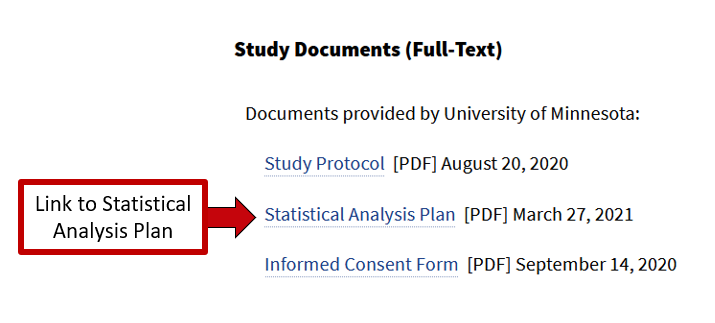


# **3. Datasets Data**

## **A. Plan to share data**

**Do the researchers state they plan to share their data in their "Plan to Share IPD" in the "Individual Participant Data (IPD) Sharing Statement" section of the CTG record?**

- **Y: Yes** (The researchers state "yes" in their "Plan to Share IPD" in the CTG record)
- **N: No** (The researchers state "no" in their "Plan to Share IPD" in the CTG record)
- **U: Undecided** (The researchers state "undecided" in their "Plan to Share IPD" in the CTG record)
- **I: Irrelevant** (There is no "Individual Participant Data (IPD) Sharing Statement" in the CTG record)

**Example from NCT04386616 in CTG**

Link: [https://classic.clinicaltrials.gov/ct2/show/NCT04386616](https://classic.clinicaltrials.gov/ct2/show/NCT04386616?term=NCT04386616&draw=2&rank=1)


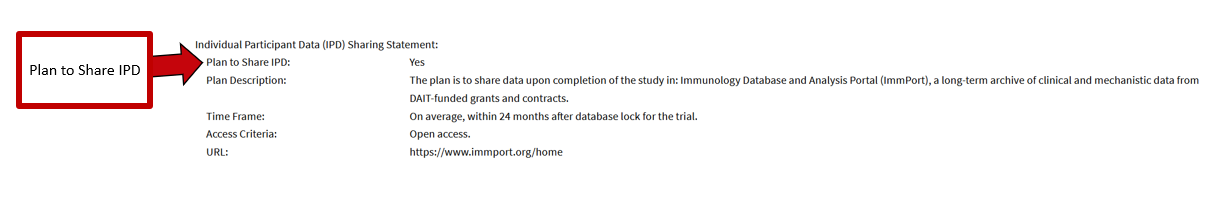


## **B. Plan description/access criteria**

**Do the researchers state they plan to share their data with non-affiliates in either their "Plan Description" or their "Access Criteria" in the "Individual Participant Data (IPD) Sharing Statement" section of the CTG record?**

**Note:** Researchers must state they plan to share data, either identified or deidentified, in either the "Plan Description" or "Access Criteria" in order to be categorized as a "yes." Plans that specify that they will only share summary data or genetic sequencing data will be categorized as a "no." Statements that data will be only shared within the research team/affiliated institution(s) will also result in a "no" categorization.

- **Y: Yes** (The researchers state they plan to share their data in the "Plan Description" and/or "Access Criteria" in the CTG record)
- **N: No** (The researchers state they do not plan to share their data in the "Plan Description" or "Access Criteria" in the CTG record)
- **U: Undecided** (The researchers state they are undecided on whether to share their data in the "Plan Description" or "Access Criteria" in the CTG record)
- **I: Irrelevant** (There is no "Plan Description" nor "Access Criteria" in the CTG record)

**Example from NCT04386616 in CTG**

Link: [https://classic.clinicaltrials.gov/ct2/show/NCT04386616](https://classic.clinicaltrials.gov/ct2/show/NCT04386616?term=NCT04386616&draw=2&rank=1)


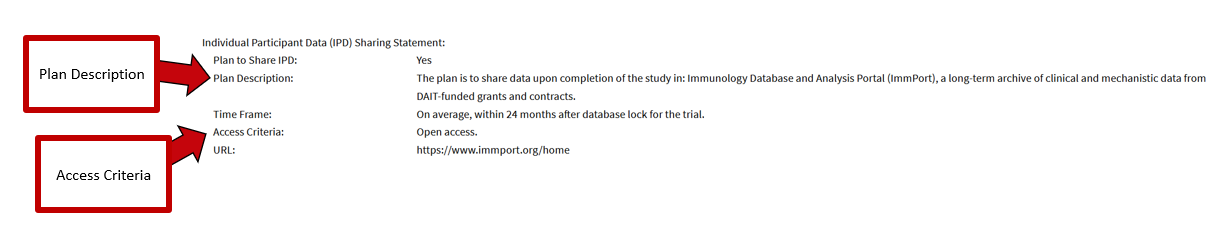


## **C. Plan inconsistencies**

**Is there an inconsistency between the "Plan to Share IPD" and "Plan Description"/"Access Criteria" in the CTG record? E.g., "No" for the "Plan to Share IPD," "Yes" for the "Plan Description"**

- **Y: Yes** (There are inconsistencies between the "Plan to Share IPD" and "Plan Description"/"Access Criteria" in the CTG record)
- **N: No** (There are no inconsistencies between the "Plan to Share IPD" and "Plan Description"/"Access Criteria" in the CTG record)
- **I: Irrelevant** (Either:
  - 1) there is no "Individual Participant Data (IPD) Sharing Statement" section in the CTG record, or
  - 2) there is a "Plan to Share IPD" but no "Plan Description" nor "Access Criteria" in the CTG record)

**Example from NCT04386616 in CTG (example has no inconsistencies)**

Link: [https://classic.clinicaltrials.gov/ct2/show/NCT04386616](https://classic.clinicaltrials.gov/ct2/show/NCT04386616?term=NCT04386616&draw=2&rank=1)


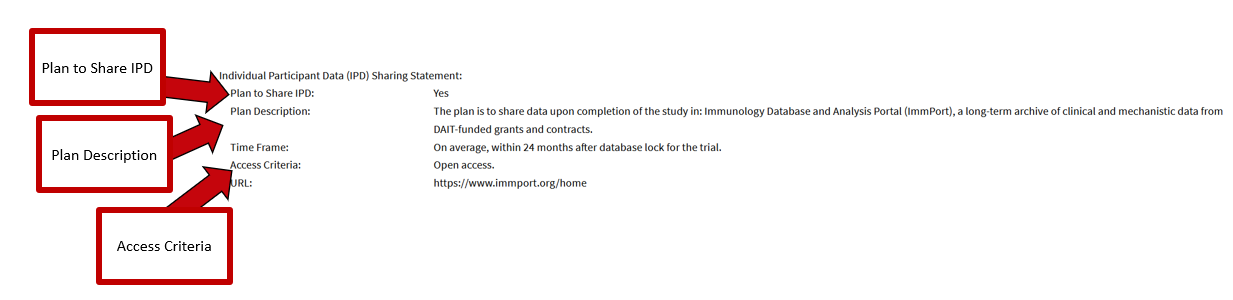


## **D. Timeframe**

**What is the timeframe for sharing data listed in the "Time Frame" in the "Individual Participant Data (IPD) Sharing Statement" section of the CTG record?**

- **[Textual entries for specified timeframes]**
- **U: Unspecified** (The researchers state "yes" in their "Plan to Share IPD" and/or "Plan Description"/"Access Criteria," but do not include a timeframe for sharing their data in the "Time Frame" in the CTG record)
- **I: Irrelevant** (Either:
  - 1) there is no "Individual Participant Data (IPD) Sharing Statement" in the CTG record, or
  - 2) the researchers state "no" or "undecided" in their "Plan to Share IPD" and in their "Plan Description"/"Access Criteria" in the CTG record, or
  - 3) the researchers state "no" or "undecided" in their "Plan to Share IPD" and don't have a "Plan Description" nor "Access Criteria" in the CTG record)

**Example from NCT04386616 in CTG**

Link: [https://classic.clinicaltrials.gov/ct2/show/NCT04386616](https://classic.clinicaltrials.gov/ct2/show/NCT04386616?term=NCT04386616&draw=2&rank=1)


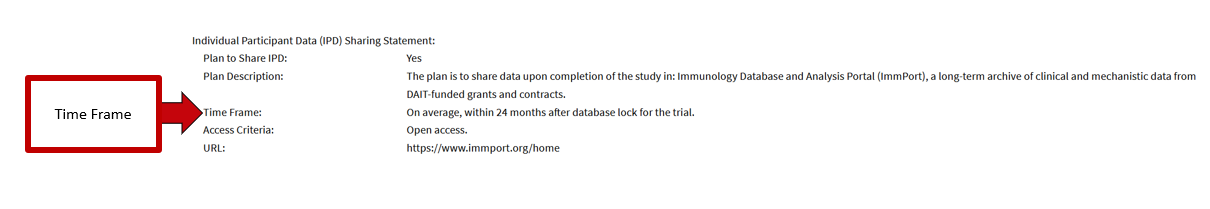


## **E. Mechanism for getting access to data**

**What is the mechanism for getting access to the data listed in either the "Plan Description" or the "Access Criteria" in the "Individual Participant Data (IPD) Sharing Statement" section of the CTG record?**

**Note:** Mechanisms for accessing summary data or genetic sequencing data will not be included (i.e., only mechanisms for accessing IPD are included). Mechanisms by which *only* the research team(s) or affiliated institution(s) can access data will also be excluded (but will be mentioned in the notes section) (i.e., only mechanisms for unaffiliated researchers accessing data will be included).

- **P: Data sharing platform** (The "Plan Description" and/or the "Access Criteria" states requestors may access the data via a data sharing platform)
- **R: Researcher contact** (The "Plan Description" and/or the "Access Criteria" states requestors must contact the PI/researcher to get access to the data. This also includes statements of "data can be accessed upon request" when a platform isn't mentioned)
- **C: Could not determine** (The CTG record states "yes" in their "Plan to Share IPD" and/or in their "Plan Description"/"Access Criteria", but the CTG record does not mention the mechanism for getting access to the data)
- **I: Irrelevant** (Either:
  - 1) there is no "Individual Participant Data (IPD) Sharing Statement" section in the CTG record, or
  - 2) the researchers state "no" or "undecided" in their "Plan to Share IPD" and in their "Plan Description"/"Access Criteria" in the CTG record, or
  - 3) the researchers state "no" or "undecided" in their "Plan to Share IPD" and have no "Plan Description" nor "Access Criteria" in the CTG record)

**Example from NCT04386616 in CTG**

Link: [https://classic.clinicaltrials.gov/ct2/show/NCT04386616](https://classic.clinicaltrials.gov/ct2/show/NCT04386616?term=NCT04386616&draw=2&rank=1)


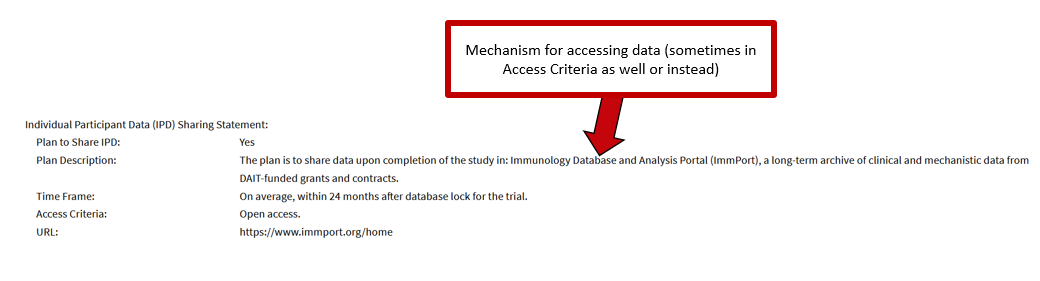


## F. Email address provided

**In cases where researcher contact is listed as the mechanism for getting access to data in either the "Plan Description" or the "Access Criteria" in the "Individual Participant Data (IPD) Sharing Statement" section of the CTG record, is an email address provided anywhere in the CTG record?**

- **Y: Yes** (An email address is provided in the CTG record)
- **N: No** (An email address is not provided in the CTG record)
- **I: Irrelevant** (Either:
  - 1) there is no "Individual Participant Data (IPD) Sharing Statement" section in the CTG record, or
  - 2) researcher contact is not listed as a mechanism for getting access to the data in the "Plan Description" nor the "Access Criteria" in the CTG record)

**Example from NCT04364737 in CTG**

Link: [https://classic.clinicaltrials.gov/ct2/show/NCT04364737](https://classic.clinicaltrials.gov/ct2/show/NCT04364737?term=NCT04364737&draw=2&rank=1)


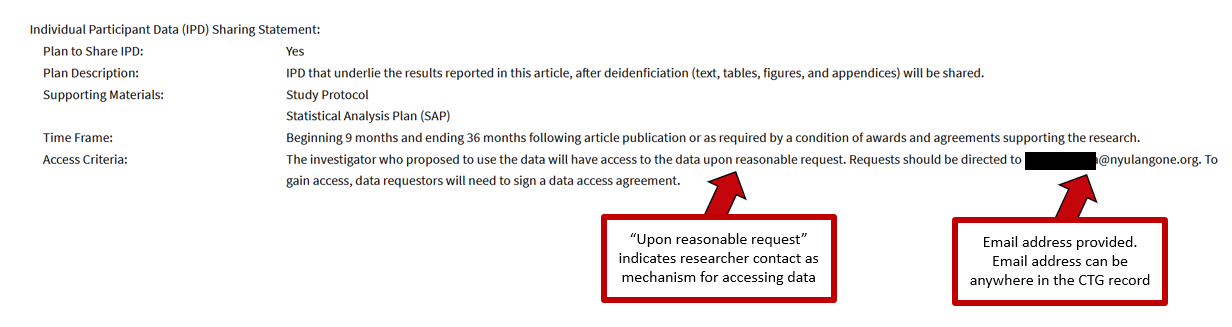


## **G. Platform name provided**

**Is the name of the data sharing platform in which the researchers plan to share their data listed in the either the "Plan Description" or the "Access Criteria" in the "Individual Participant Data (IPD) Sharing Statement" section of the CTG record?**

- **Y: Yes** (The name of the data sharing platform in which the researchers plan to share their data is listed in the "Plan Description" and/or the "Access Criteria" in the CTG record)
- **N: No** (The CTG record states requestors may access the data via a data sharing platform in the "Plan Description" and/or "Access Criteria," but neither the "Plan Description" nor the "Access Criteria" list the name of the data sharing platform in which the researchers plan to share their data)
- **I: Irrelevant** (Either:
  - 1) there is no "Individual Participant Data (IPD) Sharing Statement" section in the CTG record, or
  - 2) data sharing platform is not listed as a mechanism for getting access to the data in the "Plan Description" nor the "Access Criteria" in the CTG record)

**Example from NCT04386616 in CTG**

Link: [https://classic.clinicaltrials.gov/ct2/show/NCT04386616](https://classic.clinicaltrials.gov/ct2/show/NCT04386616?term=NCT04386616&draw=2&rank=1)


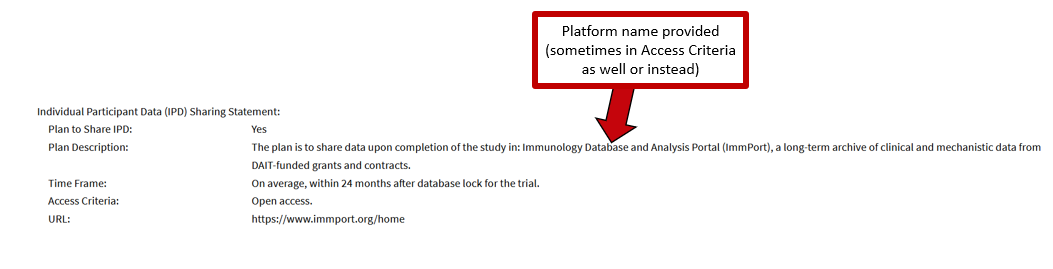


## H. Discoverability

**For datasets shared on a data sharing platform, how discoverable is the trial's associated dataset record?**

**Note:** For trials that named a specific data sharing platform as a mechanism for sharing data in either the "Plan Description" or the "Access Criteria" in the "Individual Participant Data (IPD) Sharing Statement" section of the CTG record, but didn't provide a direct link to the dataset record, PW searched the platform for the dataset record using the trial's NCT number or, if the latter retrieved no results, searched the platform using the trial's title in its corresponding CTG record

- **D: Direct** (There is a direct, functional link to the dataset record present in the "Individual Participant Data (IPD) Sharing Statement" section of the CTG record)
- **A: Additional searching required** (PW had to search the data sharing platform mentioned in the "Plan Description" and/or the "Access Criteria" of the CTG record to locate the dataset record)
- **N: Not discovered** (A specific data sharing platform was mentioned in the "Plan Description" and/or the "Access Criteria" in the CTG record, but PW was unable to locate the dataset record in the platform)
- **P: Platform is private or otherwise inaccessible** (A specific data sharing platform was mentioned in the "Plan Description" and/or the "Access Criteria" in the CTG record, but PW couldn't search for the dataset record because the platform was inaccessible)
- **I: Irrelevant** (Either:
  - 1) there is no "Individual Participant Data (IPD) Sharing Statement" section in the CTG record, or
  - 2) data sharing platform is not listed as a mechanism for getting access to the data in the "Plan Description" nor the "Access Criteria" in the CTG record, or
  - 3) no platform name was mentioned and no link to the dataset record was present in the "Plan Description" nor the "Access Criteria" in the CTG record)

**Example from NCT04386616 in CTG (note: no CTG records with a direct link to a data record were found in this sample)**

Link: [https://classic.clinicaltrials.gov/ct2/show/NCT04386616](https://classic.clinicaltrials.gov/ct2/show/NCT04386616?term=NCT04386616&draw=2&rank=1)


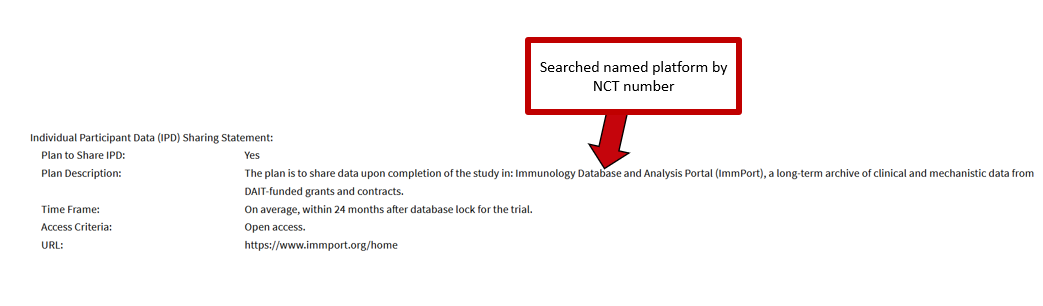


## I. Link back to CTG

**For CTG records were an associated dataset was found in a data sharing platform, is there a link from the dataset record back to the CTG record?**

- **Y: Yes** (There is a link back to the CTG record present in the dataset record)
- **N: No** (There is no link back to the CTG record present in the dataset record)
- **I: Irrelevant** (The dataset record is unavailable or could not be located)

**Example from Vivli for NCT04386616**

Link: <https://search.vivli.org/studyDetails/fromSearch/c25397f3-9256-404c-8262-dc56636afd7b>


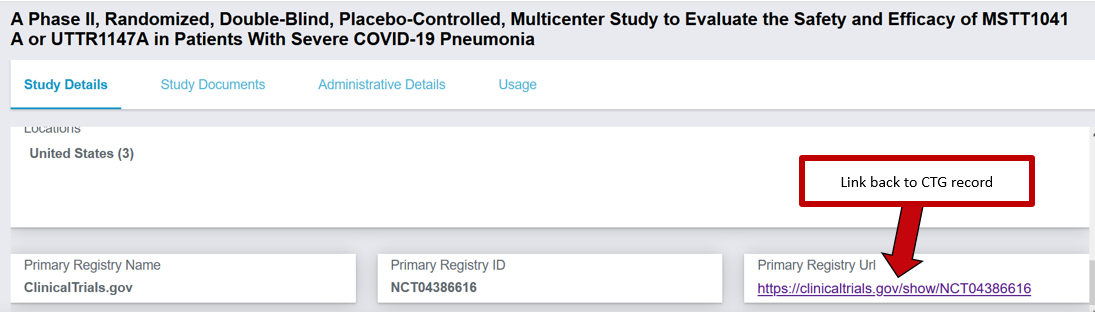


# **4. Publications Data**

**Note:** In this context, publication record refers to a publication record in PubMed .

## **A. Link to publication in CTG record**

**Link to the publication record is listed in the "More Information" section of the ClinicalTrials.gov (CTG) record**

- **[Link to publication record]**
- **[Blank]** (no link to a publication record was listed in the "More Information" section of the CTG record)

**Example from NCT04660422 in CTG**

Link: [https://classic.clinicaltrials.gov/ct2/show/NCT04660422](https://classic.clinicaltrials.gov/ct2/show/NCT04660422?term=NCT04660422&draw=2&rank=1)


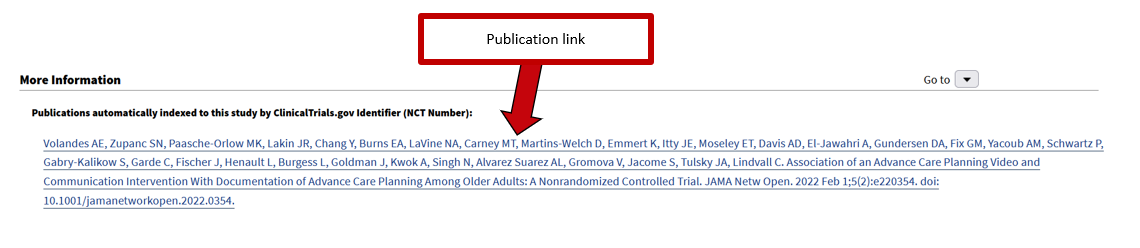


## **B. Automatically linked**

(Was the linked publication record automatically indexed into the "More Information" section of the CTG record?)

- **Y: Yes** (the linked publication record was automatically indexed into the CTG record)
- **N: No** (the linked publication record was manually added into the CTG record)
- **I: Irrelevant** (no link to a publication record was listed in the "More Information" section of the CTG record)

**Example from NCT04660422 in CTG**

Link: [https://classic.clinicaltrials.gov/ct2/show/NCT04660422](https://classic.clinicaltrials.gov/ct2/show/NCT04660422?term=NCT04660422&draw=2&rank=1)


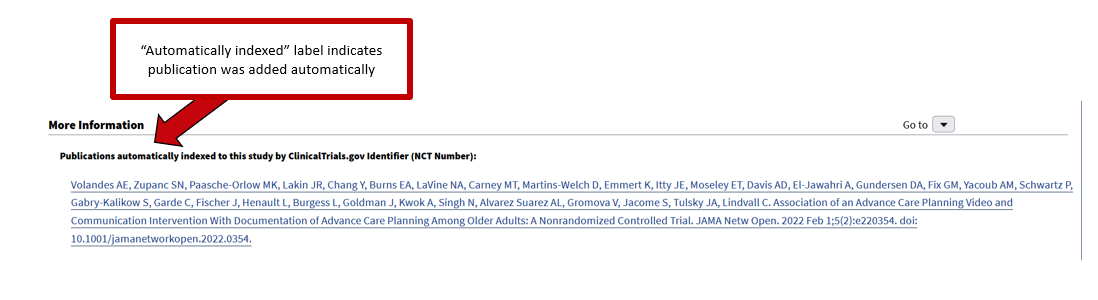


## **C. Link type (for manually added publication record links)**

**For publication record links added manually to the CTG record, what was their categorization when using the XML view in the CTG record?**

**Note:** The XML view is accessible by adding **?resultsxml=true** to the end of the CTG record link

- **C: Citation** (the field code <reference> is assigned to the publication record link)
- **R: Result** (the field code <results reference> is assigned to the publication record link)
- **I:** **Irrelevant** (either:
  - 1) the publication record link was added automatically to the CTG record, and thus doesn't have a categorization, or
  - 2) no link to a publication record was listed in the "More Information" section of the CTG record)

**Example from NCT04355767 in CTG**

Link: [https://classic.clinicaltrials.gov/ct2/show/NCT04355767](https://classic.clinicaltrials.gov/ct2/show/NCT04355767?term=NCT04355767&draw=2&rank=1)


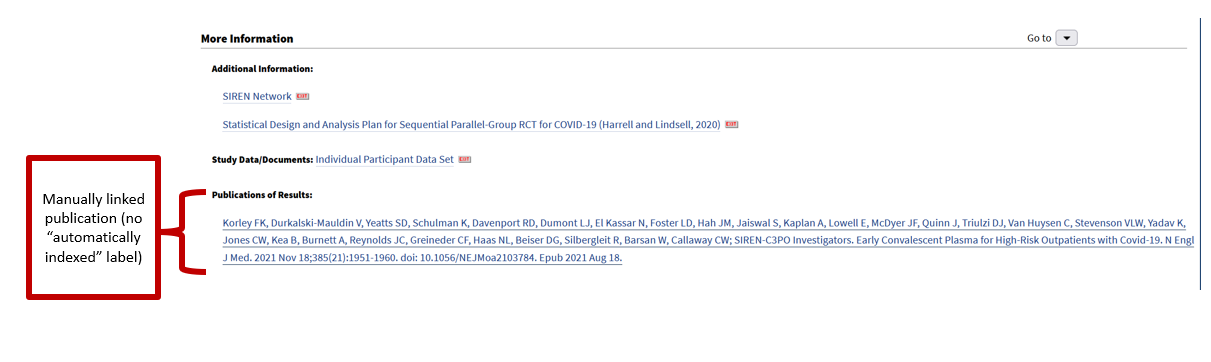


**Non-results XML view of manually linked publication example from NCT04706403 in CTG**

Link: <https://classic.clinicaltrials.gov/ct2/show/NCT04706403?term=NCT04706403&draw=2&rank=1?resultsxml=true>


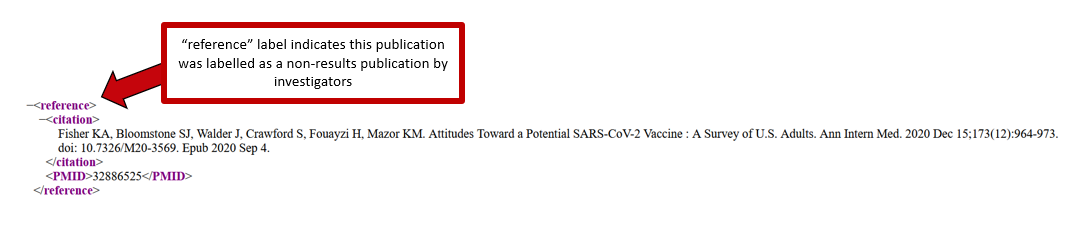


**Results XML view of manually linked publication example from NCT04355767 in CTG**

Link: <https://classic.clinicaltrials.gov/ct2/show/NCT04355767?term=NCT04355767&draw=2&rank=1?resultsxml=true>


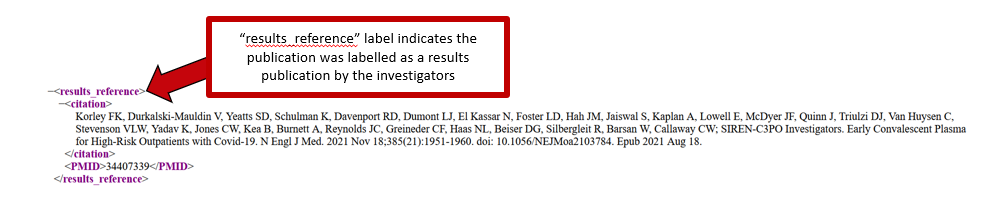


## **D. PubMed [si] test**

**Was the publication record retrieved when searching the NCT number with the [si] field tag in PubMed? (E.g., NCT04363203[si])**

- **Y: Yes** (the publication record was retrieved by a [si] search)
- **N: No** (the publication record was not retrieved by a [si] search)

## **E. PubMed [tw] test**

**Was the publication record retrieved when searching the NCT number with the [tw] field tag in PubMed? (E.g., NCT04363203[tw])**

- **Y: Yes** (the publication record was retrieved by a [tw] search)
- **N: No** (the publication was not retrieved by a [tw] search)

## **F. PMID**

**PMID of the publication record linked in the "More Information" section of the CTG record**

- **[PMID]**
- **[Blank]: Irrelevant** (there was no publication record link in the "More Information" section of the CTG record)

**Example from PubMed:**

Link: <https://pubmed.ncbi.nlm.nih.gov/34224257/>


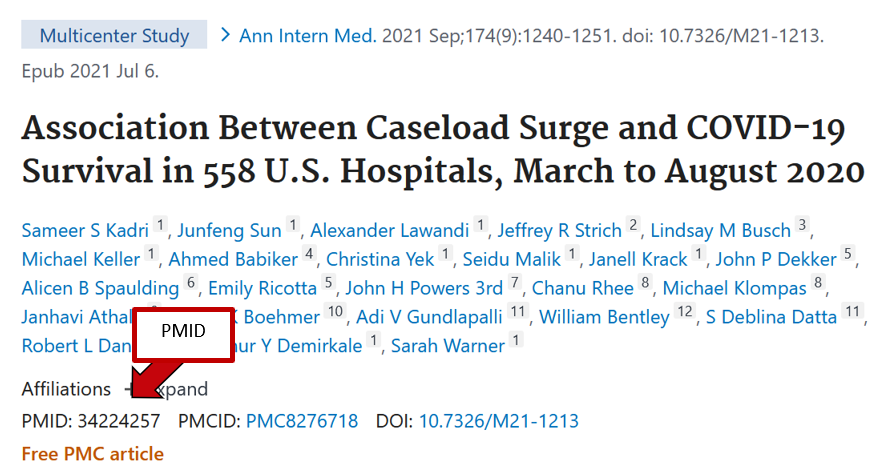


## **G. NCT number included somewhere in the publication record**

**Was the NCT number included somewhere in the publication record associated with the CTG record?**

- **Y: Yes** (the NCT number was included in the publication record)
- **N: No** (the NCT number was not included in the publication record)
- **I: Irrelevant** (no link to a publication record was listed in the "More Information" section of the CTG record and no publication record was found using the [si] nor [tw] search)

**Example from publication record in PubMed:**

Link: <https://pubmed.ncbi.nlm.nih.gov/34224257/>


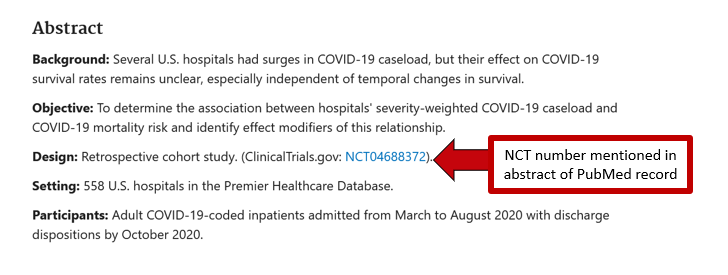


## **H. Associated data link back to CTG record**

**Did the publication record have an "Associated Data" link back to the CTG record?**

- **Y: Yes** (the publication record had an "Associated Data" link back to the CTG record)
- **N: No** (the publication record did not have an "Associated Data" link back to the CTG record)
- **I: Irrelevant** (no link to a publication record was listed in the "More Information" section of the CTG record and no publication record was found using the [si] nor [tw] search)

**Example from publication record in PubMed (towards the bottom of the record in PubMed)**

Link: <https://pubmed.ncbi.nlm.nih.gov/34224257/>


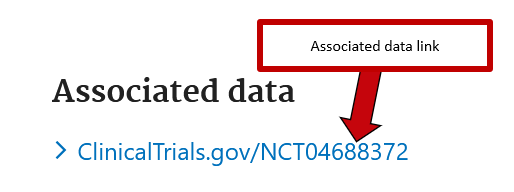


## **I. LinkOut link back to CTG record**

**Did the publication record have a "LinkOut" link back to the CTG record?**

- **Y: Yes** (the publication record had a "LinkOut" link back to the CTG record)
- **M: Link to multiple CTG records** (the publication record had a "LinkOut" link to a page containing multiple CTG records, one of which was the CTG record of interest)
- **N: No** (the publication record did not have a "LinkOut" link back to the CTG record)
- **I: Irrelevant** (no link to a publication record was listed in the "More Information" section of the CTG record and no publication record was found using the [si] nor [tw] search)

**Example from publication record in PubMed (towards the bottom of the record in PubMed)**

Link: <https://pubmed.ncbi.nlm.nih.gov/34224257/>


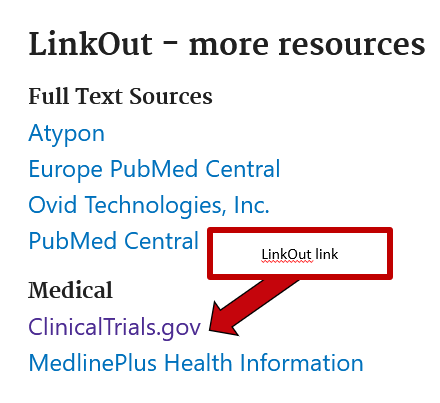


## **J. Abstract link back to CTG record**

**Did the publication record have a link in its abstract back to the CTG record?**

- **Y: Yes** (the publication record had a link in its abstract back to the CTG record)
- **N: No** (Either
  - 1) the publication record did not have a link in its abstract back to the CTG record, or
  - 2) the publication record did not have an abstract)
- **I: Irrelevant** (no link to a publication record was listed in the "More Information" section of the CTG record and no publication record was found using the [si] nor [tw] search)

**Example from publication record in PubMed**

Link: <https://pubmed.ncbi.nlm.nih.gov/34224257/>


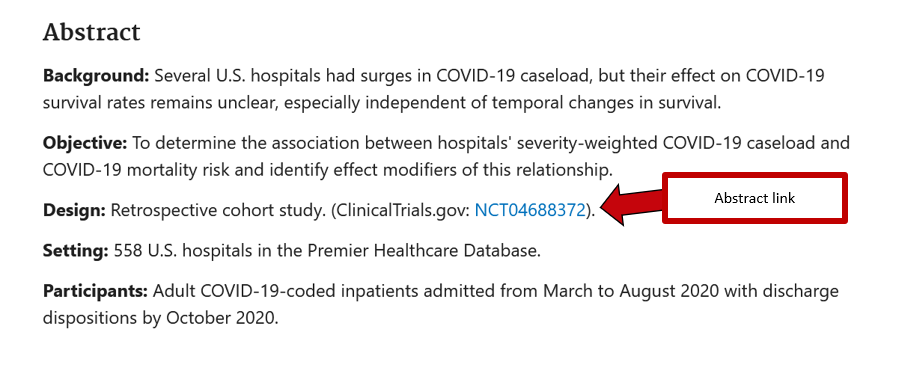

Supplement: Supplementary file 2 — Appendix B [file jmla-112-3-250-s02.docx]
