# Supplementary material for: Evaluating the discoverability of supporting research materials in ClinicalTrials.gov for US federally funded COVID-19 clinical studies
Source: J Med Libr Assoc. 2024 Jul 29;112(3):250–60. doi: 10.5195/jmla.2024.1799 (PMC11412123; doi:10.5195/jmla.2024.1799)
Supplement: Supplementary file 3 — Appendix C [file jmla-112-3-250-s03.pdf]

Appendix C: Results Verification Decision Tree

Flow Chart

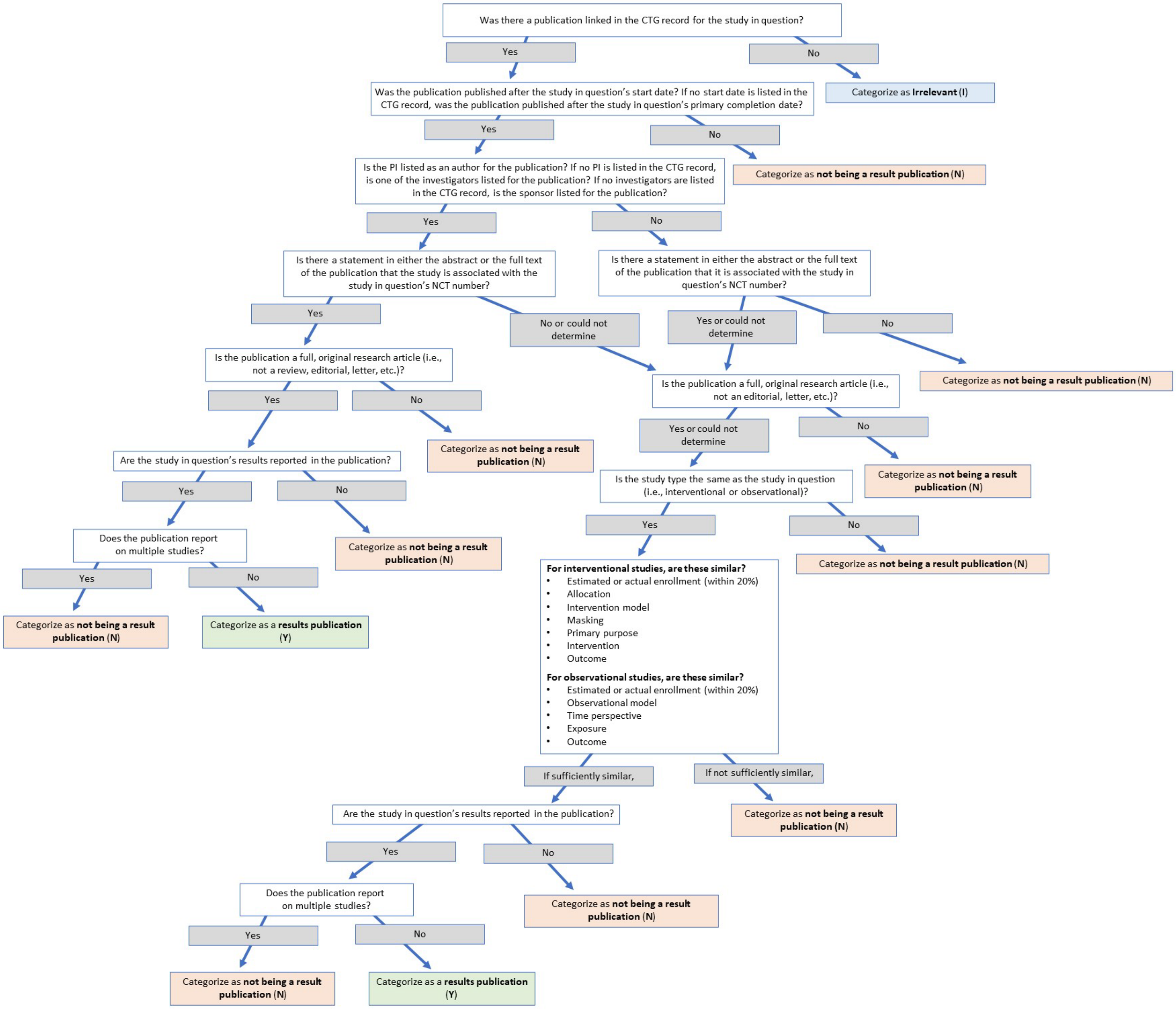

Narrative Version of Flow Chart

1. **Was there a publication linked in the CTG record for the study in question?** If no, categorize as irrelevant (I). If yes, move to 2.
2. **Was the publication published after the study in question's start date? If no start date is listed in the CTG record, was the publication published after the study in question's primary completion date?** If no, categorize as not being a result publication (N). If yes, move on to 3.
3. **Is the PI listed as an author for the publication? If no PI is listed in the CTG record, is one of the investigators listed for the publication? If no investigators are listed in the CTG record, is the sponsor listed for the publication?** If no, move on to 3a. If Yes, move to 3b.
    a. **Is there a statement in either the abstract or the full text of the publication that it is associated with the study in question's NCT number?** If no categorize as not being a result publication (N). If yes or could not determine move to 3ai
        i. **Is the publication a full, original research article (i.e., not an editorial, letter, etc.)?** If no categorize as not being a result publication (N). If yes or could not determine move to 3aii
        ii. **Is the study type the same as the study in question (i.e., interventional or observational)?** If no categorize as not being a result publication (N). If yes move to 3aiii
        iii. **For interventional studies, are these similar?:** Estimated or actual enrollment (within 20%), Allocation, Intervention model, Masking, Primary purpose, Intervention, Outcome. **For observational studies, are these similar?:** Estimated or actual enrollment (within 20%), Observational model, Time perspective, Exposure, Outcome. If not sufficiently similar, categorize as not being a result publication (N). If sufficiently similar, move to 3aiv
        iv. **Are the study in question's results reported in the publication?** If no, categorize as not being a result publication (N). If yes, move to 3av
        v. **Does the publication report on multiple studies?** If no categorize as a result publication (Y). If yes, categorize as not being a result publication (N).
    b. **Is there a statement in either the abstract or the full text of the publication that the study is associated with the study in question's NCT number?** If no or could not determine, move to 3Ai. If yes move to 3bi
        i. **Is the publication a full, original research article (i.e., not a review, editorial, letter, etc.)?** If no, categorize as not being a result publication (N). If yes, move to 3bii
        ii. **Are the study in question's results reported in the publication?** If no categorize as not being a result publication (N). If yes move to 3biii
        iii. **Does the publication report on multiple studies?** If no categorize as being a result publication (Y). If yes categorize as not being a result publication (N).
