# Supplementary material for: Evaluating the discoverability of supporting research materials in ClinicalTrials.gov for US federally funded COVID-19 clinical studies
Source: J Med Libr Assoc. 2024 Jul 29;112(3):250–60. doi: 10.5195/jmla.2024.1799 (PMC11412123; doi:10.5195/jmla.2024.1799)

Appendix IV: Characteristics of Sample

# Table 1

This table summarizes characteristics of the study records exported from ClinicalTrials.gov on June 6, 2022, which were used for the study. A subset of the data is also shown containing characteristics of study records that had a completed status.

| **CTG Records' Characteristics** | | |
| --- | --- | --- |
| **Status (N=206)** | | |
| Recruiting | 96 | (47%) |
| Completed | 42 | (20%) |
| Active, not recruiting | 40 | (19%) |
| Not yet recruiting | 14 | (7%) |
| Enrolling by invitation | 9 | (4%) |
| Terminated | 3 | (1%) |
| Suspended | 2 | (1%) |
| **Study Type (N=206)** | | |
| Interventional | 110 | (53%) |
| Observational | 96 | (47%) |
| **Phases of Interventional Trials (N=110)** | | |
| Not Applicable | 54 | (49%) |
| Phase 1 | 0 | (0%) |
| Phase 1\|Phase 2 | 2 | (2%) |
| Phase 2 | 18 | (16%) |
| Phase 2\|Phase 3 | 5 | (5%) |
| Phase 3 | 26 | (24%) |
| Phase 4 | 5 | (5%) |
| **Characteristics of Completed Trials** | | |
| **Study Type (N=42)** | | |
| Interventional | 23 | (55%) |
| Observational | 19 | (45%) |
| **Phases of Interventional Trials (N=23)** | | |
| Not Applicable | 8 | (35%) |
| Phase 1 | 0 | (0%) |
| Phase 1\|Phase 2 | 0 | (0%) |
| Phase 2 | 5 | (22%) |
| Phase 2\|Phase 3 | 0 | (0%) |
| Phase 3 | 10 | (43%) |
| Phase 4 | 0 | (0%) |

# Figure 1

This bar chart shows the ClinicalTrials.gov (CTG) records by start year. "Completed Status" refers to records marked as "Completed" in CTG. "Incomplete Status" refers to all other study status types (i.e., all records *not* marked as "Completed"). Most records had a start date between 2020 and 2021.


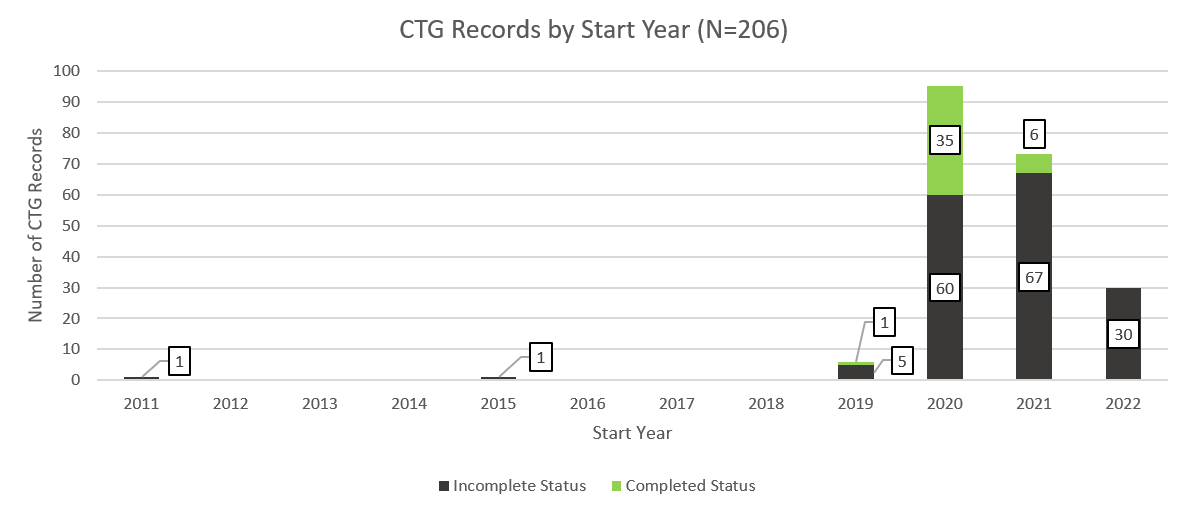


# Figure 2

The bar chart below shows the number of CTG records with an incomplete status (i.e., records *not* marked as "Completed") that did or didn't share enough information to request access to IPD by start year. Records were classified as having shared enough information to request access to IPD if they shared *both* a mechanism by which they planned to share IPD with unaffiliated researchers (i.e., via email or data sharing platform) and at least one email address or the name of the data sharing platform, where applicable. Only 11% of records provided enough information to request access to IPD. The majority of these had start dates of 2021 and 2022.


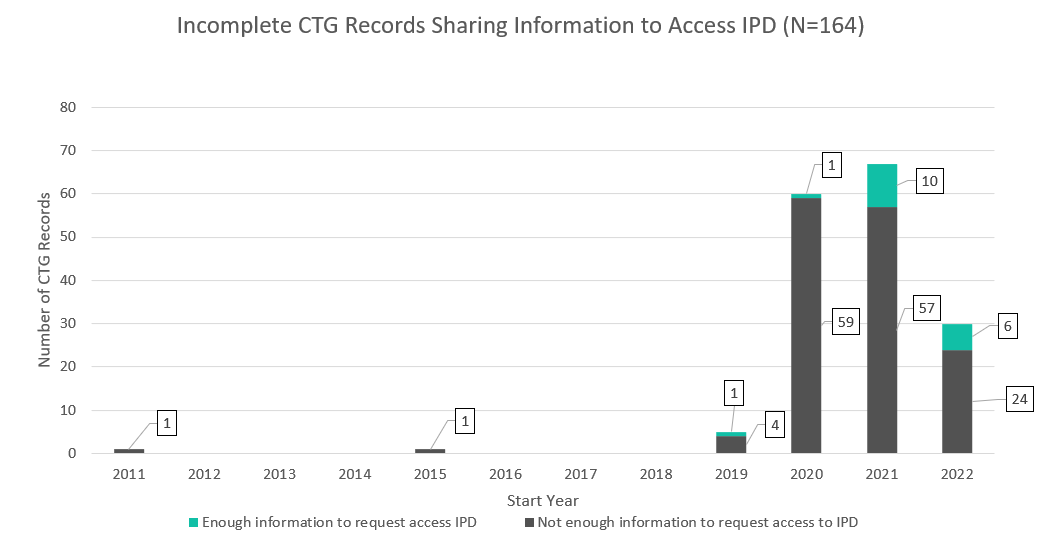


# Figure 3

The bar chart below shows the number of CTG records marked as "Completed" that did or didn't share enough information to request access to IPD by start year. Records were classified as having shared enough information to request access to IPD if they shared *both* a mechanism by which they planned to share IPD with unaffiliated researchers (i.e., via email or data sharing platform) and at least one email address or the name of the data sharing platform, where applicable. Only 17% provided enough information to request access to IPD, with the majority of these records having a start date of 2020.


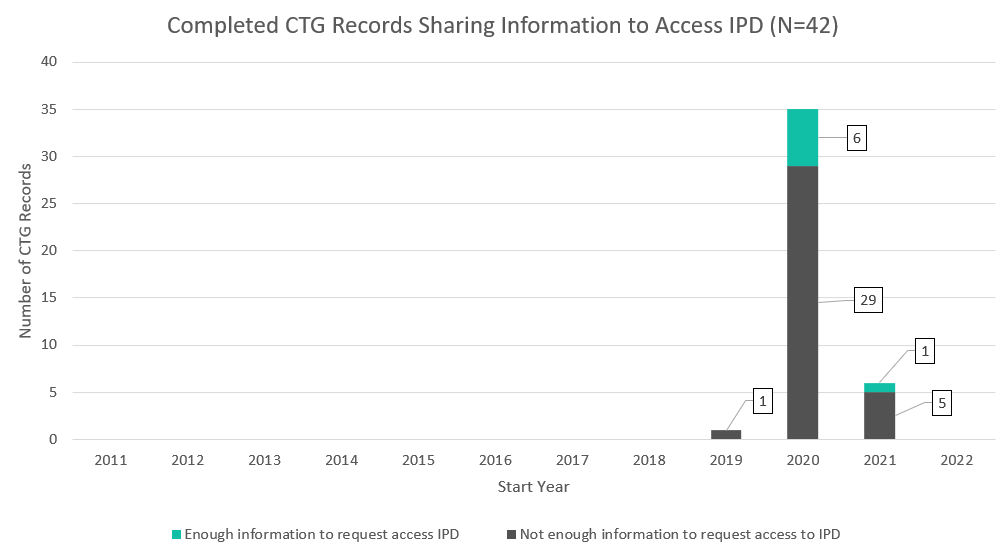


# Figure 4

The bar chart below shows the number of incomplete CTG records (i.e., all records *not* marked as "Completed" in their status) that linked out to at least 1 results publication by start year. Most records did not link out to a results publication, and those that did mostly had a 2020 or 2021 start date.


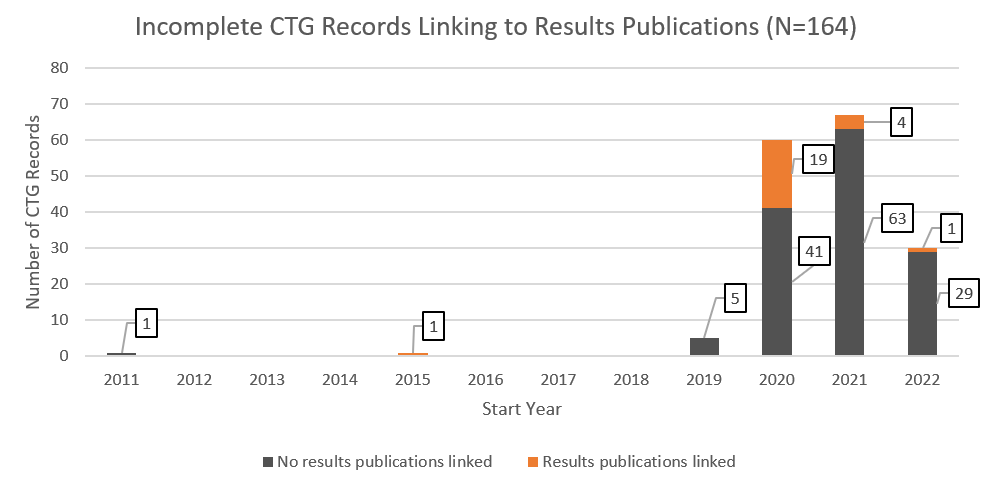


# Figure 5

The bar chart below shows the number of CTG records marked as "Completed" that did or did not link to at least 1 results publication by start year. A little less than half of the records linked to results publication records, with the ones that did having a start date of 2020.


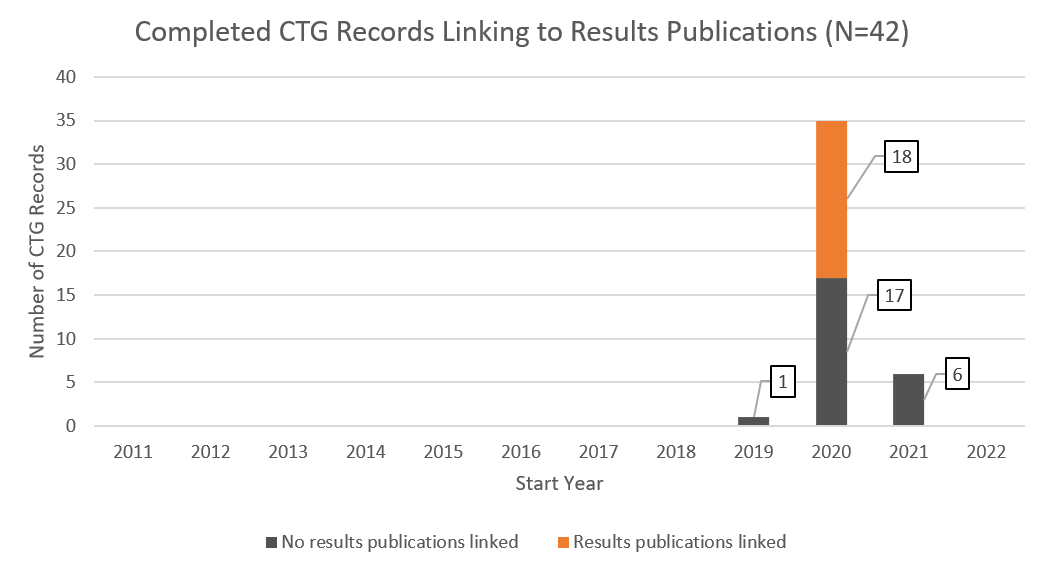

Supplement: Supplementary file 4 — Appendix D [file jmla-112-3-250-s04.docx]
